# Characterization of the Xiamenmycin Biosynthesis Gene Cluster in *Streptomyces xiamenensis* 318

**DOI:** 10.1371/journal.pone.0099537

**Published:** 2014-06-11

**Authors:** Yong Yang, Ling Fu, Jinlong Zhang, Linghan Hu, Minjuan Xu, Jun Xu

**Affiliations:** 1 State Key Laboratory of Microbial Metabolism and School of Life Sciences. & Biotechnology, State Key Laboratory of Ocean Engineering, Shanghai Jiao Tong University, Shanghai, China; 2 Key Laboratory of Systems Biomedicine, Shanghai Center for Systems Biomedicine, Shanghai Jiao Tong University, Shanghai, China; 3 Institute of oceanology, Shanghai Jiao Tong University, Shanghai, China; INIAV, I.P.- National Institute of Agriculture and Veterinary Research, Portugal

## Abstract

Xiamenmycin (**1**) is a prenylated benzopyran derivative with anti-fibrotic activity. To investigate the genetic basis of xiamenmycin biosynthesis, we performed genome mining in the xiamenmycin-producing *Streptomyces xiamenensis* wild-type strain 318 to identify a candidate gene cluster. The complete gene cluster, consisting of five genes, was confirmed by a series of gene inactivations and heterologous expression. Based on bioinformatics analyses of each gene and feeding experiments, we found that the structure of an intermediate xiamenmycin B (**3**) accumulated in a *ximA* inactivation mutant, allowing us to propose a biosynthetic pathway. All five of the genes in the pathway were genetically and biochemically characterized. XimA was biochemically characterized as an ATP-dependent amide synthetase, catalyzing an amide bond formation in the presence of ATP as the final step in Xiamenmycin biosynthesis. The *K*_m_ value of XimA was determined to be 474.38 µM for the substrate xiamenmycin B. These studies provide opportunities to use genetic and chemo-enzymatic methods to create new benzopyran derivatives as potential therapeutic agents.

## Introduction

Unlike plants, microorganisms form very few natural prenylated products as secondary metabolites [1]. Among those that are produced, benzopyran and its derivatives generally have low cellular toxicity and good membrane permeability [2]. One example is xiamenmycin (**1**), which in 2000 was reported to be an inhibitor of ICAM-1/LFA-1 interaction with a possible anti-inflammatory function [3]. Another report from 2012 found that xiamenmycin not only blocks the adhesion of monocytes to lung fibroblasts but also inhibits the contractile capacity of lung fibroblasts [4]. More recently, it was found that xiamenmycin can attenuate hypertrophic scar formation in a mechanical stretch-induced mouse model [4]. Therefore, xiamenmycin is a promising agent for treating fibrotic diseases. *Streptomyces xiamenensis* 318, which was originated from mangroves [5], was used to produce xiamenmycin. However, the biosynthetic gene cluster responsible for producing prenylated benzopyran derivatives remains unknown.

The chemical structure of xiamenmycin can be divided into three parts: l-threonine, 4-hydroxybenzoic acid (4HB) and a geranyl group. Known as the key intermediate in the biosynthesis of ubiquinone, 4HB is derived from chorimate by chorismate lyase. In 1974, an *E. coli* mutant deficient in 4HB synthesis was isolated [6], and the *ubi*C gene, which encodes chorimate lyase, was cloned and sequenced in 1992 [7], [8]. The biochemical characterization, the reaction mechanism and the crystal structure of chorimate lyase were subsequently reported [9], [10].

The membrane-bound 4HB oligoprenyltransferase (UbiA) is a key enzyme in ubiquinone biosynthesis that catalyzes the prenylation of 4HB. The *ubi*A gene was cloned and sequenced in 1992 [8], [11], and a structural model of UbiA from *E. coli* was later produced [12], [13]. The biochemical characterizations of UbiAs from *Lithospermum erythrorhizon* and *E. coli* have been attempted [14]–[16]. It has been reported that UbiA could participate in the biosynthesis of microbial secondary metabolites, such as aurachin alkaloids [17]. Based on the structural features of xiamenmycin, a prenyltransferase was thought to play a key role in the prenylation of 4HB and could thus be used as a target for screening the xiamenmycin biosynthetic gene cluster.

In this paper, we describe a gene cluster consisting of five genes that is responsible for the biosynthesis of **1** and propose a biosynthetic pathway for **1**. We show that 4-Hydroxybenzoic acid is the first intermediate for **1** biosynthesis. Through biochemical characterization, we also demonstrate that XimC is responsible for the generation of 4HB. XimB catalyzes 4HB and geranyl diphosphate (GPP) to produce 3-geranyl-4-hydroxybenzoic acid (**2**). The prenylated 4HB is then processed by XimD to generate an epoxide intermediate, followed by catalysis of pyran ring formation by XimE, a SnoaL-like polyketide cyclase, to generate xiamenmycin B (**3**). Finally, XimA was biochemically characterized to be responsible for catalyzing the amide formation of **3** and L-threonine to produce **1**.

## Results

### Identification and Verification of the Biosynthetic Gene Cluster of 1 in *S. xiamenensis* 318

The 5.9 M bp draft genome sequence (unpublished data) of *S. xiamenensis* 318, which produces **1**, was annotated using the RAST server (http://rast.nmpdr.org/). From this analysis, we identified six homologues of 4-hydroxybenzoate polyprenyltransferase (UbiA), which might catalyze prenylation of 4HB during ubiquinone biosynthesis [18]. Transcription of three *ubi*A genes (ORF4925, ORF5065, ORF5313) was confirmed using real-time reverse-transcription-PCR (data not shown).

One of the *ubi*A genes was thought to be located in the gene cluster responsible for biosynthesis of xiamenmycin. The DNA fragment containing both the *ubi*A gene and a putative chorismate lyase gene that is responsible for generating 4-Hydroxybenzoic acid was chosen for further characterization.

We constructed a genomic library of *S. xiamenensis* 318 in *Escherichia coli* using the fosmid vector pCC2FOS (Table S1). One fosmid (p9A11), which has been shown to cover the complete biosynthetic gene cluster, was obtained by PCR screening. Subcloning of a 7.5 kb DNA fragment from p9A11 generated the plasmid pLMO09403, which contained five open reading frames (ORF5311, ORF5313, ORF5314, ORF5315, ORF5316) used for further genetic analysis (Table 1).

**Table 1:** Deduced ORFs and their predicted functions in the *xim* gene cluster.

Gene	Size (aa)^a^	Proposed function	Protein homolog^b^	Accession No.	Protein similarity/identity, (%/%)
*ximA* (ORF5311)	520	amide synthetase	putative substrate-CoA ligase	WP_009721027.1	94/89
*ximB* (ORF5313)	313	4-hydroxybenzoate geranyltransferase	Putative 4-hydroxybenzoate polyprenyltransferase	WP_009721026.1	92/90
*ximC* (ORF5314)	196	chorismate lyase	hypothetical protein	un-annotated ORF	(86/78)^c^
*ximD* (ORF5315)	473	epoxidase	secreted protein	WP_009721025.1	94/89
*ximE* (ORF5316)	124	SnoaL-like cyclase	hypothetical protein	WP_009721024.1	94/92

a aa, amino acids.

b genome annotation based on *Streptomyces himastatinicus* ATCC 53653 whole genome shotgun sequence cont1.771.

c DNA sequence identity of 86% was observed in the un-annotated ORF in *Streptomyces himastatinicus* ATCC 53653 cont1.771, whole genome shotgun sequence.

To verify the involvement of this DNA fragment in the biosynthesis of **1**, five gene replacement plasmids were constructed and introduced to *S. xiamenensis* 318. We individually replaced *xim*A (ORF5313), *ximB* (ORF5311), *ximC* (ORF5314), *ximD* (ORF5315), and *ximE* (ORF5316) with an apramycin resistance cassette (see Experimental Section for details). These mutants were confirmed by comparing the sizes of PCR products using the primers listed (Table S2).

**Figure 1:**
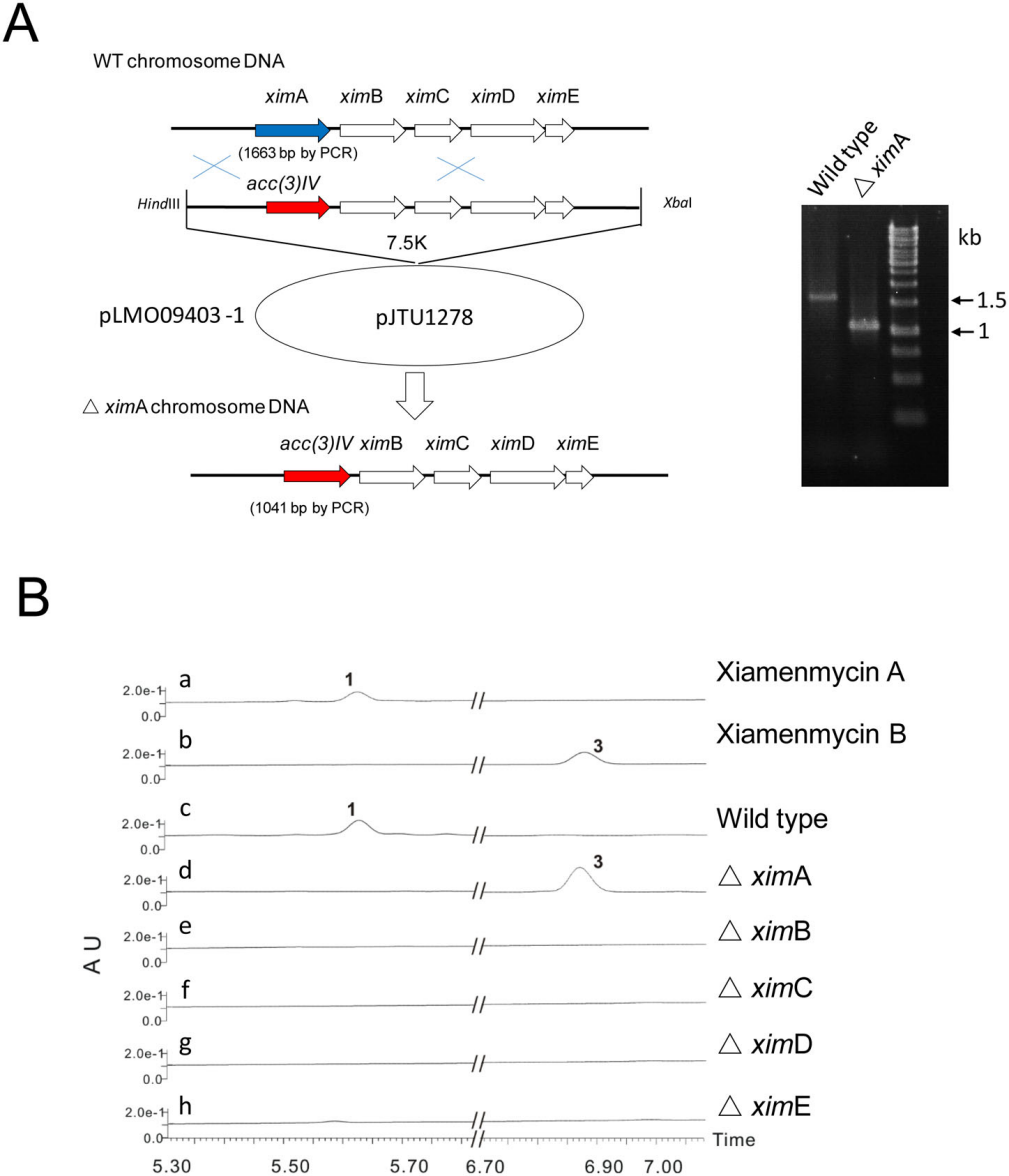
Mutational analysis of the xiamenmycin gene cluster. (A) Schematic representation of the construction of the gene inactivation mutant (*ximA*), where *ximA* was replaced by the *apr* cassette. The *ximA* mutant gives a 1041 bp PCR product, while the wild type strain is 1663 bp. Marker (1 kb DNA Ladder, Fermantas). (B) UPLC profiles of 1, 3 and products of wild type and xim mutants. Disruption of ximA to ximE individually abolished production of 1, but the intermediate 3 only accumulated in ΔximA.

**Figure 2:**
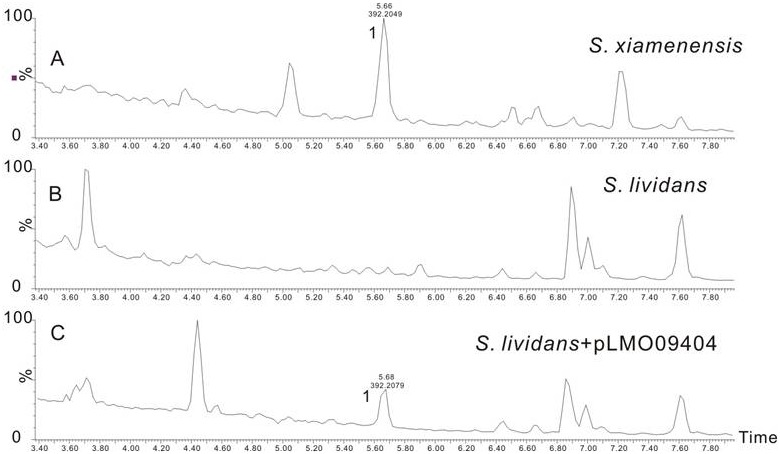
Heterologus expression of the xiamenmycin biosynthetic pathway in *S. lividans*. UPLC- total ion chromatography MS profiles of the metabolites produced by (A) *S. xiamenensis* wild type; (B) *S. lividans* harboring the empty vector pSET152; (C) *S. lividans* containing pLMO09404.

Subsequently, the gene disruption mutants were investigated for the production of **1** and its related derivatives by UPLC (Ultra Performance Liquid Chromatography). This analysis revealed that ximA inactivation mutants produced an intermediate (**3**) instead of **1** (Figure 1), while **1** production was abolished in the other four gene disruption mutants without accumulation of detectable intermediate. **3** was purified by reverse-phase semi-preparative HPLC (See Experimental Section). Further analysis of ^1^H and ^13^C NMR, as well as two-dimensional NMR spectra data, confirmed the structure of **3** to be 3-hydroxy-2-methyl-2-(4-methylpent-3-enyl)chroman-6-carboxylic acid (Table S3–S4 and Figure S1–S7). Heterologous expression of the biosynthetic gene cluster described above in *S. lividans* 1326 was then attempted. The secondary metabolite profile of the resulting *S. lividans* exconjugant was analyzed by HPLC and UPLC-Q-TOF-MS, using wild type *S. xiamenensis* 318 and *S. lividans* 1326 harboring empty pSET152 vector as control strains. In contrast to controls, the integrated gene cluster enabled *S. livdans* 1326 to produce **1** (Figure 2). These results suggested that, as expected, introduction of five genes (*ximA*, *ximB*, *ximC*, *ximD*, and *ximE*) into S. livdans 1326 was sufficient for formation of **1**; however, their respective functions remained unclear.

### Proposed Biosynthetic Pathway for Xiamenmycin

Bioinformatics analysis revealed a high sequence similarity between XimA and many proteins dependent on CoA, such as a substrate-CoA ligase from *Streptomyces himastatinicus* (89% identity), a long-chain-fatty-acid-CoA ligase from *Amycolatopsis azurea* (44% identity), and an AMP-dependent synthetase and ligase from *Streptomyces* sp. CNS615 (43% identity). However, none of these enzymes has been functionally characterized. In contrast, we found that XimA displays relatively low amino acid sequence similarity to the typical acyl CoA synthetase from *E. coli* (26% identity). A conserved domain search of XimA showed that it contains the Class I adenylate-forming domain present in FadD [19]. This domain catalyzes an ATP-dependent two-step reaction to first activate a carboxylate substrate as an adenylate and then transfer the carboxylate to the phosphopantetheinyl group of either coenzyme A or a holo acyl-carrier protein. This family includes acyl- and aryl-CoA ligases, as well as the adenylation domain of nonribosomal peptide synthetases. However, we assumed that XimA was an amide synthetase rather than a substrate-CoA ligase, catalyzing the amide formation of l-threonine with the carboxyl group of the intermediate **3** that accumulated in the Δ*ximA* mutants.

In the database, many XimB homologues were annotated as a 4-hydroxybenzoate polyprenyltransferases. Sequence coverage of over 90% and 50% identity between XimB and the top ten hits (BlastP, E-value<1e-81) suggested that these homologues belong to the so-called UbiA superfamily. Therefore, similar to UbiA, XimB was predicted to catalyze a prenylation of 4HB.

No hits were found using BlastP against the Refseq database with XimC as the querying sequence, but 87% DNA sequence identity was observed with an un-annotated ORF in *S. himastatinicus* ATCC 53653 cont1.771. Although XimC displays no identity with the typical UbiC from *E. coli*, it shares almost 30% amino acid sequence identity with the putative chorismate pyruvate-lyase in *Methylococcus capsulatus* (E-value = 0.0027) and *Pseudomonas putida* (E-value = 0.15), providing a hint that XimC could catalyze the conversion of chorismate to 4HB.

XimD showed high sequence similarity to many FAD-binding proteins. A conserved domain search of XimD showed that it contains UbiH [20] multi-domains present in 2-polyprenyl-6-methoxyphenol hydroxylase and other related FAD-dependent oxidoreductase. XimD contains the geranylgeranyl reductase family multi-domains, which are usually involved in chlorophyll and bacteriochlorophyll biosynthesis. This result suggested that the function of XimD could be to catalyze an epoxidation reaction to generate an epoxide intermediate.

XimE showed high sequence similarity to three hypothetical proteins, including one each from *S. himastatinicus* (92% identity), *Streptomyces griseoaurantiacus* (59% identity), and *Streptomyces* sp. R1-NS-10 (51% identity). However, none of these enzymes has been functionally characterized. A conserved domain search of XimE showed that it contains a specific SnoaL-like domain present in the polyketide cyclase (SnoaL) involved in nogalamycin biosynthesis [21]. SnoaL belongs to a family of small polyketide cyclases and catalyzes the ring closure steps in the biosynthesis of polyketide antibiotics produced in *Streptomyces* [21]. We therefore hypothesized that XimE could catalyze a pyran ring formation.

**Figure 3:**
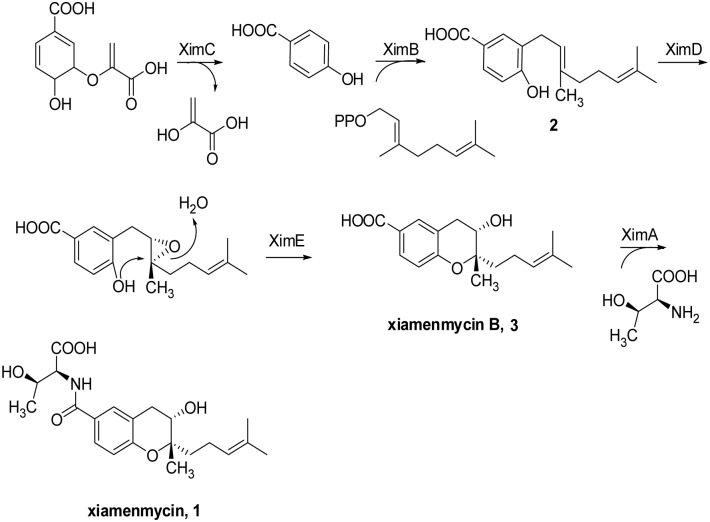
Proposed biosynthetic pathway for xiamenmycin.

On the basis of the structure of **3** and the bioinformatics analysis of *ximA*, *ximB*, *ximC*, *ximD*, and *ximE*, we proposed a biosynthetic pathway for xiamenmycin, as depicted in Figure 3. The pathway starts with the formation of 4HB by the putative chorismate lyase encoded by *ximC*. The linkage of the geranyl side chain to the benzene nucleus is most likely then catalyzed by the gene product of *ximB*. XimD, an epoxidase, may generate an epoxide intermediate. XimE, a SnoaL-like cyclase, could catalyze the pyran ring formation concomitant with the opening of this epoxide intermediate to produce **3**. The final amide bond formation is likely catalyzed by XimA.

### The Function of *Xim*C is to Produce 4HB

**Figure 4:**
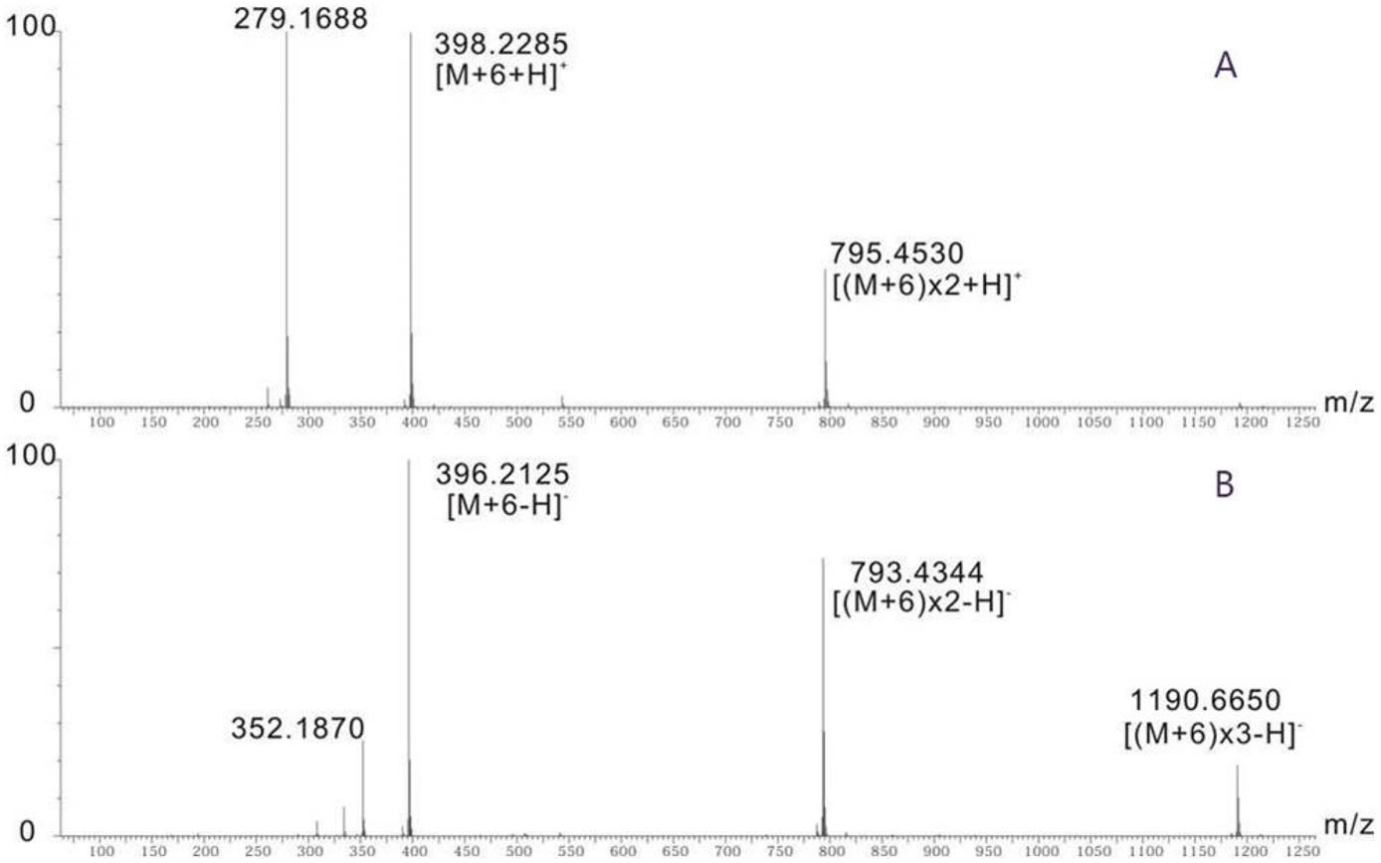
Confirmation of 4HB in the core structure of xiamenmycin. HR-MS of **1** after [ring-^13^C_6_] 4HB was fed to *S. xiamenensis* wild type strain, (A) in the positive ionization mode; (B) in the negative ionization mode.

**Figure 5:**
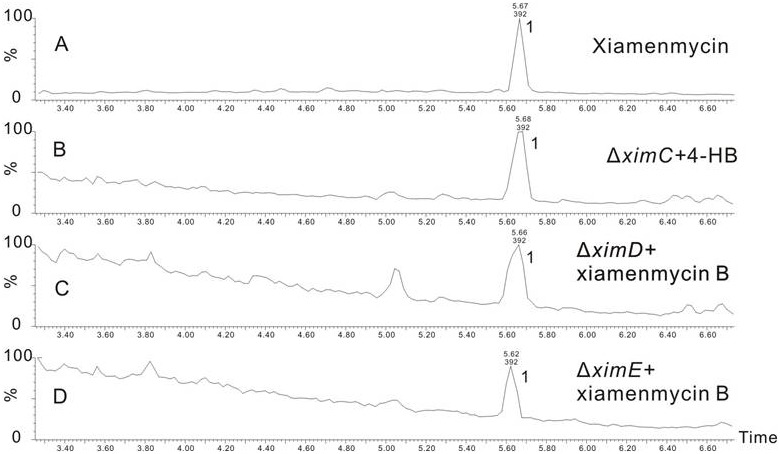
*In vitro* assay of XimC. GC-MS profiles from the *in* *vitro* assay of XimC. (A) 4HB; (B) heat-inactivated XimC incubated with chorismate; (C) XimC incubated with chorismate.

XimC shows low homology to the putative chorismate pyruvate-lyase in *M. capsulatus* and *P. putida*. The inactivation of *ximC* completely abolished the production of **1**, while supplementing [ring-^13^C_6_] 4HB by feeding restored **1** production (see Figure 4). This result suggests that XimC may be a chorismate lyase, catalyzing decomposition of chorismate to generate 4HB as the first step of Ximenmycin biosynthesis. We subsequently overexpressed and purified N-terminally His_6_-tagged XimC from *E. coli* BL21 (DE3). When the purified XimC protein (Figure S8A) was incubated with chorismate, the formation of 4HB was observed and confirmed by GC-MS (Figure 5). As a negative control, heat-inactivated XimC was incubated with chorismate, leading to only trace amounts of 4HB formation due to chemical decomposition of chorismate [9]. Comparing the amount of 4HB generated *in* *vitro* by XimC to the amount formed in the negative control with heat-inactivated XimC, we confirmed that XimC is indeed a chorismate lyase that catalyzes cleavage of chorismate to produce 4HB and pyruvate [9].

### The Function of *Xim*B is to Produce 2

XimB displayed 34% identity with the biochemically characterized *E. coli* UbiA (4-hydroxybenzoate:polyprenyldiphosphate 3-polyprenyltransferase) [22], which prenylates 4HB with GPP. The SOSUI program (http://bp.nuap.nagoya-u.ac.jp/sosui) predicted that XimB contains twelve putative transmembrane helices.

**Figure 6:**
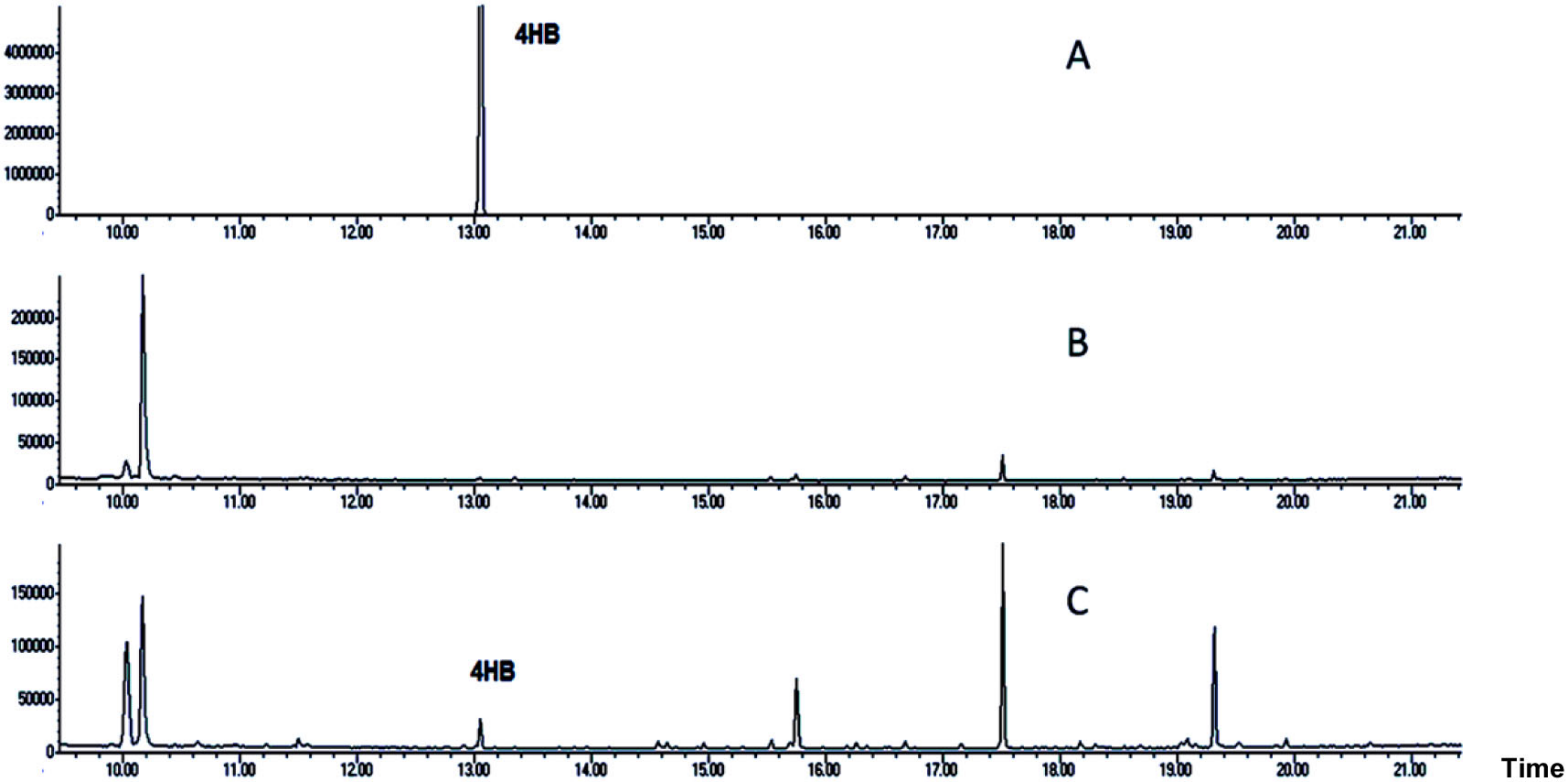
*In vitro* assay of XimB. UPLC-total ion chromatography MS in the negative ionization mode of the XimB *in* *vitro* assay. (A) HR-MS/MS of compound **2**; the lines display the proposed permutations and combination pattern; (B) UPLC-total ion chromatography MS of the membrane fraction without XimB incubated with 4HB and GPP; (C) UPLC-total ion chromatography MS of XimB incubated with 4HB and GPP.

When the membrane fraction containing XimB was incubated with 4HB and GPP in the presence of Mg^2+^, a substantial amount of product **2** was observed and confirmed by MS/MS analysis (Figure 6). As a negative control, the membrane fraction without XimB was also incubated with 4HB and GPP in the presence of Mg^2+^. This assay resulted in the production of trace amounts of **2** due to contaminated UbiA from *E. coli* in the membrane fraction. Comparing the amounts of **2** produced *in* *vitro* by XimB and the negative control suggested that the membrane protein XimB is a 4-hydroxybenzoate geranyltransferase, which could utilize 4HB and GPP to produce **2**.

However, when the membrane fraction containing XimB was incubated with thirteen other 4HB analogues (Figure S9) in the presence of GPP and Mg^2+^, or with 4HB in the presence of Mg^2+^ and dimethylallyl diphosphate (DMAPP) or farnesyl diphosphate (FPP), no prenylated products were detected (data not shown). In addition, we attempted to supplement the media with a group of 4HB analogues (Figure S9), including 4-aminobenzoic acid, 4-mercaptobenzoic acid and others to feed Δ*xim*C mutant; however, no detectable prenylated products were produced (data not shown). Therefore, XimB seemed to only utilize 4HB and GPP as substrates for producing prenylated products.

### XimA as an Amide Synthetase for Amide Bond Formation

**Figure 7:**
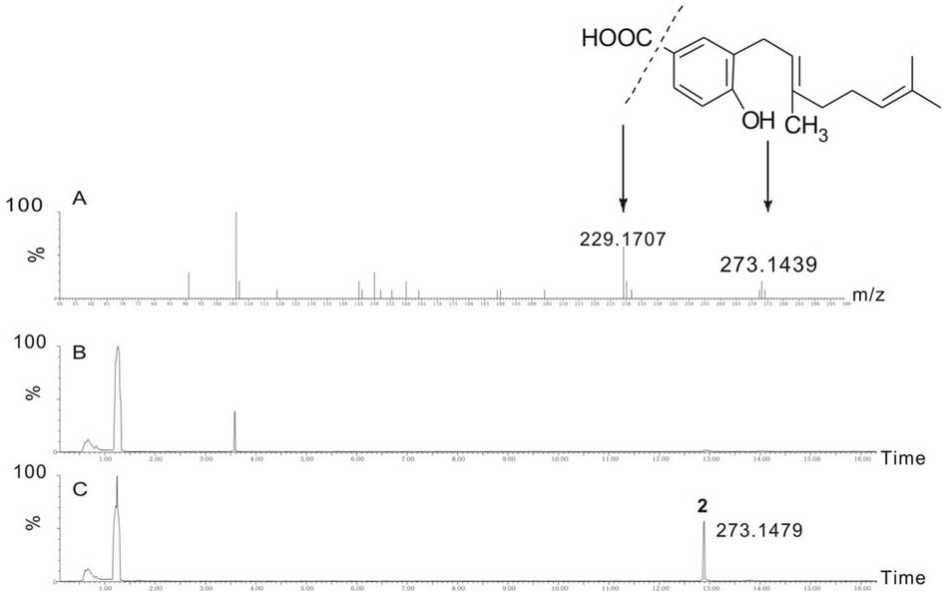
Feeding experiments of the *xim* gene disruptants. UPLC- total ion chromatography MS profiles of extracts of feeding experiments. (A) xiamenmycin (**1**); (B) Δ*ximC*, supplemented with 0.1 mg/ml 4HB; (C) Δ*ximD* mutant supplemented with 0.1 mg/ml xiamenmycin B (**3**); (D) Δ*ximE* mutant supplemented with 0.1 mg/ml xiamenmycin B (**3**).

Accumulation of **3** was only detected in the Δ*ximA* mutant. According to the chemical structures of **3** and **1**, we deduced that pyran ring formation occurs before the amide bond formation catalyzed by XimA. When **3** was added into the medium at a final concentration of 0.1 mg/ml, the production of **1** was restored in both Δ*ximD* and Δ*ximE* mutants (Figure7). These data indicate that XimA catalzyes amide bond formation as the final step in the biosynthesis of xiamenmycin.

**Figure 8:**
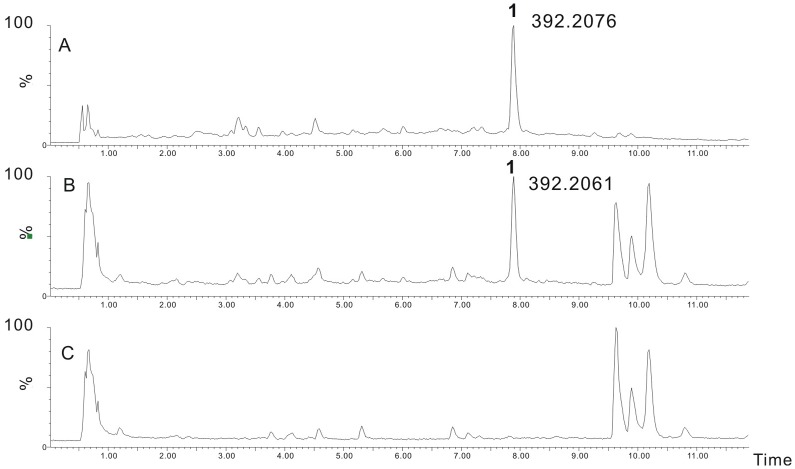
*In vitro* assay of XimA. UPLC-extracted ion chromatography MS (EIC-MS) in the negative ionization mode of XimA *in vitro* assay. (A) **xiamenmycin** (**1**); (B) XimA incubated with **xiamenmycin B** (**3**) and L-threonine; (C) boiled XimA incubated with **xiamenmycin B** (**3**) and L-threonine.

XimA shows the highest homology to acyl- or aryl- CoA ligases or adenylation domains of non-ribosomal peptide synthetases, which catalyze a two-step reaction. Fatty acids, aromatic acids, or amino acids were activated in their adenylated forms in the presence of ATP. Activated acyl, aryl or aminoacyl was then transferred to the thiol group of CoA or holo peptidyl carrier proteins. Therefore, we hypothesized that XimA may act as an ATP-dependent amide synthetase that catalyzes the amide bond formation mediated by ATP. XimA was overexpressed and purified from *E. coli* as an N-terminally His_6_-tagged protein (Figure S8B). When the purified XimA protein was incubated with **3**, L-threonine, and ATP, the product **1** was observed (Figure 8). In contrast, when the reaction was carried out with heat-inactivated XimA no product was detected. Therefore, *ximA* may be coding for an amide synthetase, which could utilize **3** and L-threonine to produce **1**. In addition, when we tried to add nineteen other kinds of l- amino acids into the medium to feed the *S. xiamenensis* wild type strain, no amidation products were detected (data not shown).

Therefore, XimA was biochemically confirmed to be an ATP-dependent amide synthetase utilizing **3** and l-threonine as substrates for amide bond formation. The *K*_m_ value of XimA for xiamenmycin B was determined to be 474.38 µM (Figure S10).

## Discussion

Our study reported a gene cluster that is involved in 1 biosynthesis in *S. xiamenensis* 318. Using a series of gene inactivations and heterologous expression, we found this gene cluster to consist of five ORFs. On the basis of the structure of the accumulated compound, feeding studies, biochemical characterizations, and bioinformatics analysis of each gene, we proposed the putative biosynthetic pathway of 1 that was featured in pyran ring formation.

The first and the second step of the xiamenmycin biosynthetic pathway were analogous to the well-studied biosynthesis of ubiquinones [18]. The high substrate specificity of XimB for 4HB and GPP was not consistent with the relaxed substrate tolerance of UbiA in ubiquinone biosynthesis, but similar to the low substrate tolerance of the homologous UbiA involved in shikonin biosynthesis [14], [22].

The structural difference between the final product **1** and the intermediate **3** suggests that the amino acid moiety was loaded onto the core structure by XimA after closing of the benzopyran ring. XimA included conserved domains responsible for AMP and CoA binding that have commonly been characterized as a substrate-CoA ligase of the Class I adenylate-forming superfamily. This family includes acyl- and aryl-CoA ligases, as well as the adenylation domain of nonribosomal peptide synthetases. The adenylate-forming enzymes catalyze an ATP-dependent two-step reaction to first activate a carboxylate substrate as an adenylate and then transfer the carboxylate to the phosphopantetheine group of either coenzyme A or an acyl-carrier protein. However, when the purified XimA protein was incubated with **3** and l-threonine in the presence of CoA, no acylated products were observed (data not shown). Thus, XimA only utilize **3** and L-threonine as substrates for amide bond formation.

Biochemical characterizations of benzopyran ring formation are rarely reported because of the scarcity of benzopyran derivatives as secondary metabolites. Moreover, the existence of a ring 3′-OH makes the catalytic mechanism different from that of ring formation catalyzed by Fe^3+^ [2] or chalcone isomerase [23]–[32]. We hypothesized that an oxidative cyclization catalyzed by XimD and XimE are plausible.

To test this hypothesis, we overexpressed and purified XimD and XimE in *E. coli* BL21 (DE3) (Figure S8C). As proposed above, product **2** (Figure 3) of XimB should be the substrate of XimD and XimE; therefore, the purified XimD and XimE were incubated with the membrane fraction containing XimB, 4HB and GPP in the presence of Mg^2+^ for *in* *vitro* production of **2**. As anticipated, **2** and the expected product **3** were observed and confirmed by LC-MS analysis (Figure S11). However, when the purified XimD and XimE were incubated with the substrates and the protein mentioned above in the presence of FAD, FMN, NAD, or NADP, only the product **2** was observed (data not shown). Furthermore, when the purified XimD and XimE were individually incubated with the membrane fraction containing XimB, 4HB and GPP in the presence of Mg^2+^, the product **3** was not observed (data not shown). XimD shows similarity (33%) to LasC, which catalyzes the epoxide formation in lasalocid biosynthesis [33], so we propose that XimD may also catalyze a similar epoxide formation. Subsequently, XimE catalyzes a nucleophilic attack of a phenolic hydroxyl group to the epoxide to ultimately form the pyran ring.

## Conclusion

In summary, the putative xiamenmycin biosynthetic pathway starts with the formation of 4HB by XimC. The linkage of the geranyl side chain to the benzene nucleus is catalyzed by XimB. XimD, an epoxidase, may generate an epoxide intermediate, and XimE, a SnoaL-like cyclase, could catalyze this epoxide intermediate to produce **3**. The subsequent amide bond formation is likely to be catalyzed by XimA. A similar gene cluster was identified in the draft genome sequence of *S. himastatinicus* ATCC 53653. The very high identity of each Xim protein (XimA to XimE) in *S. xiamenesis* to its counterparts in *S. himastatinicus* pave the way for exploiting combinatorial biosynthesis based on the characterized biosynthetic pathway for the generation of xiamenmycin derivatives with improved bioactivity.

## Materials and Methods

### Chemicals

Kanamycin, isopropyl β-D-1-Thiogalactopyranoside (IPTG), chloramphenicol, L-threonine, D-threonine and ampicillin were purchased from Sangon Biotech (Shanghai, China); Apramycin, nalidixic acid, CoA, chorimate, geranyl diphosphate (GPP), farnesyl diphosphate (FPP), dimethylallyl diphosphate (DMAPP), FADH_2_, FAD, FMN, NAD, NADP, thiostrepton, [ring-^13^C_6_] 4-hydroxybenzoic acid and 4-hydroxybenzoic acid were purchased from Sigma. Thiostrepton (12.5 µg/mL), ampicillin (50 mg/mL), apramycin (30–50 mg/mL), kanamycin (30–50 mg/mL), chloramphenicol (35 mg/mL) and nalidixic acid (25 mg/mL) were used for selection of recombinant strains.

### Bacterial Strains, Plasmids and Primers

The bacterial strains and plasmids used in this study are listed in Table S1. The primers used in this study are listed in Table S2.

### Genetic Procedures

DNA extraction and manipulation in *S. xiamenensis* were performed following the protocol described by Kieser et al. [34]. DNA fragments were purified from agarose gels using a CopyControl Fosmid Library Production Kit (EPICENTRE Biotechnologies). Isolation of fosmids and plasmids was carried with ion-exchange columns (Plasmid Mini kit; OMEGA).

### Genome Sequencing and Annotation

Genome sequencing of *S. xiamenensis* was performed by Majorbio (Shanghai, China) with 454 FLX technology. A total of 625,536 reads were produced and assembled with Newbler (454/Roche). The genome sequence was annotated using the RAST server **(** http://rast.nmpdr.org/) and BLAST program (version 2.2.25) against the non-redundant protein database [35]. The DNA sequence of the gene cluster has been deposited into GenBank database under the accession No. KF313919.

### Construction and Screening of the Fosmid Library

Chromosomal DNA from *S. xiamenensis* was sheared into approximately 40 kb fragments, end-repaired and then ligated to the pCC2FOS vector. The ligation products were packaged using ultra-high efficiency MaxPlax Lambda Packaging Extracts (EPICENTRE Biotechnologies) and transduced into EPI300-T1R Plating Strain. PCR screening was performed for the identification of ORF5317 (downstream region of *ximE*) and ORF5310 (upstream region of *ximA*). PCR reactions were carried out with TAKARA *EX* Taq polymerase.

### Construction of Gene Inactivation Mutants

A 7.5 kb *Hind*III-*Xba*I fragment was amplified from fosmid p9A11 and cloned into pJTU1278 to generate plasmid pLMO09403 harboring the complete xiamenmycin gene cluster (Table S1) [36]. The gene replacement plasmids used in this study were constructed using PCR-Targeting by a similar strategy (Figure 1A, Figure S12–S15) according to the standard protocol.

For example, for the replacement of *ximA*, the apramycin resistance (*aac*(3)IV) cassette (approximately 0.9 kb) was amplified by PCR using the forward primer 5′-ATGAGACAGGAGCATCGGGTGGACATACCCGAGAACTTGTGGTTCATGTGCAGCTCCATC-3′ and the reverse primer 5′-TCACGTTCGAGGCGCATTCGACGCCGGATAGTGACGATG TGAGCTCAGCCAATCGACTG-3′. PCR was performed at an annealing temperature of 60°C. The amplified product was used to construct a gene replacement plasmid based on pLMO09403 through PCR-Targeting technology as described by Kieser et al. [34].

The resulting plasmid pLMO09403-1 was introduced into *S. xiamenensis* 318 by conjugation with *E. coli* ET 12567 (pUZ8002). After non-selective growth, the apramycin-resistant exconjugates that were sensitive to thiostrepton, putatively resulting from double-crossover events, were selected, and their genotype was then confirmed by PCR with the appropriate primers (Table S2).

### Real-time Reverse-Transcription-PCR in *S. xiamenesis*

*S. xiamenesis* 318 was precultured for 48 h in liquid TSB medium (100 mL). ISP 2 medium (100 mL) was then inoculated with the precultures (10 mL each). The flasks were shaken on a rotary shaker at 30°C and 220 rpm for 120 h. Cells were sampled from culture broth at 24 h, 48 h, and 72 h. Seed broth used for inoculation was set as the control point (0 h). Each sample was collected by centrifugation for 10 min at 6,000×g at ambient room temperature, and the resulting pellet was immediately frozen at −80°C. After motorized grinding with liquid nitrogen, the powder of mycelia was re-suspended with the TRI reagent-RNA/DNA/protein isolation kit (Molecular Research Center Inc., Cincinnati, OH, USA). RNA isolation was performed according to the manufacturer‘s instruction. Total RNA preparations were treated with DNase I to eliminate possible chromosomal DNA contamination. The absence of DNA contamination was confirmed by PCR, using primers corresponding to the ORF5317 gene. The primers used for RT- PCR are listed in Table S2.

### Heterologous Expression of the Biosynthetic Gene Cluster in *S. lividans* 1326

The entire xiamenmycin biosynthetic gene cluster was amplified using PCR with corresponding primers (Table S2). PCR reactions were carried out with TOYOBO KOD FX polymerase. To facilitate the subsequent cloning experiment, an additional restriction site (underlined) was incorporated into both primers. After sequence confirmation, the *Eco*RI-*Xba*I fragment (7.8 kb) was inserted into the same site of pSET152 to yield pLMO09404. The plasmid was introduced into *S. lividans* 1326.

### Production and Analysis of Secondary Metabolites

Each of the following cultures and HPLC analyses were performed in three independent experimental replicates. Exconjugants of all mutants and wild-type *S. xiamenensis* were precultured for 48 h in liquid TSB medium (100 mL) before inoculation into a production medium with a dilution factor of 10. The flasks were shaken on a rotary shaker at 30°C and 220 rpm for 120 h.

For isolation of **1**, the broth culture (100 mL) was centrifuged at 10000 rpm for 20 min., the supernatant was collected and evaporated at 50°C and the residue was redissolved in methanol (1 mL).

Extracts were analyzed by HPLC (Agilent 1200 series) with an RP-18 column (Agilent Eclipse XDB-C18; 4.6×150 mm; 5 µm). We used a flow rate of 0.5 mL/min with a linear gradient program of solvent A from 15% to 40% over 8 min, 40% to 55% over 11 min, 55% to 85% over 7 min, constant 85% acetonitrile for 4 min, and detection at 254 nm (solvent A: acetonitrile; solvent B: water/formic acid 999∶1). **1** for use as standard was kindly provided by Xiaoling Li and Zhongyuan You (Shanghai, China). The extracts were also examined at the Instrumental Analysis Center of Shanghai Jiao Tong University on a Waters ACQUITY UPLC system equipped with a binary solvent delivery manager and a sample manager, coupled with a Waters Micromass Q-TOF Premier Mass Spectrometer equipped with an electrospray interface (Waters Corporation, Milford, MA) in the positive ionization mode. Analysis by Acquity BEH C18 column (100 mm×2.1 mm, 1.7 µm; Waters, Milford, USA) was carried out at a flow rate of 0.4 mL/min with a linear gradient of solvent A from 10% to 100% in 25 min (solvent A: acetonitrile/formic acid 999∶1; solvent B: water/formic acid 999∶1). Detection was carried out at 254 nm.

### Isolation of Intermediate 3

A total of 20 Liters of broth culture from the *ximA* inactivation mutant were extracted with ethyl acetate and the residue containing **3** was purified by reverse-phase semi-preparative HPLC (C18 column, Kromasil, 10×250 mm) and eluted stepwise with a gradient of 15% to 100% acetonitrile to yield approximately 20 mg of a yellow powder.

### Protein Expression and Purification

For construction of the expression plasmid, Genes *ximA*, *ximB*, and *ximC* were amplified by using the corresponding primers (Table S2). Introduced restriction sites are underlined. All three genes were excised from vector pMD18-T with the corresponding endonucleases and ligated into vector pET28a using the same restriction sites. All of the recombinant proteins were expected to contain an N-terminal His tag.

For protein expression, *E. coli* BL 21(DE3) cells were grown in 1000 ml of LB medium supplemented with 30 µg/mL kanamycin or 50 µg/mL ampicillin at 30°C until an OD_600_ of 0.6 was reached. IPTG was added at a final concentration of 1 mM. After 6 h, the cells were harvested by centrifugation and broken by ultrasonication. A one-step purification of the recombinant His_6_-tag fusion protein by affinity chromatography with Ni-NTA agarose resin (GE Healthcare, USA) was carried out according to the manufacturer’s instruction (Table S9).

### XimC *In vitro* Assay

For determination of enzymatic activity, we used 50 µl of the reaction mixture containing 50 mM Tris-HCl buffer (pH 7.5), 25 µg chorismate and 0.6 mg purified XimC. After incubation for 30 min at 30°C, the reaction was quenched with 1 ml methanol. Protein was removed by centrifugation at 13,000 g for 10 min, and the supernatant was then evaporated at 50°C. The resulting residue was freeze-dried for 24 h and then dissolved in 1 ml organic solvent (chloroform:acetone = 1∶1). After adding 50 µl derivatization reagent (BATFA:TMCS = 99∶1), the reaction mixture was incubated at 80°C for 1 h. Reaction products were analyzed by GC-MS (Agilent, 7890A GC/5975C MS, USA) using a DB-5 MS column (30 m×0.25 mm×0.25 µm). 4HB was used as a standard. The control was assayed with the same conditions in the presence of heat-inactivated enzyme, which was prepared by boiling at 100°C for 30 min.

### XimB *In vitro* Assay

For determination of XimB enzymatic activity, we used 50 µl of the reaction mixture containing 50 mM Tris-HCl buffer (pH 7.5), 5 mM MgSO_4_, 0.3 mM GPP and 0.5 mM 4HB and 1 mg membrane fraction. For preparation of the membrane fraction see the reference [22]. After incubation at 30°C for 30 min, the reaction was quenched by adding 1 ml methanol. The membrane fraction was removed by centrifugation at 13,000 g for 10 min, and the supernatant was evaporated at 50°C. The remaining residue was freeze-dried for 24 h and then dissolved in 100 µl methanol. Enzymatic products were further analyzed by the UPLC-Q-TOF-MS method described above. The control was carried out under the same conditions with the membrane fraction from bacterial strains in the absence of IPTG during cultivation.

### XimA *In vitro* Assay

For determination of enzymatic activity, we used 100 µl of the reaction mixture containing 50 mM Tris-HCl buffer (pH 7.5), 5 mM MgSO_4_, 5 mM ATP, 10 µg 3, 10 mM L-threonine and 1 mg XimA. After incubation at 30°C for 12 h, the reaction was quenched by adding 1 ml methanol. The protein was removed by centrifugation at 13,000 g for 10 min, and the supernatant was then evaporated at 50°C. The remaining residue was freeze-dried for 24 h and then dissolved in 100 µl methanol. Enzymatic products were analyzed by UPLC-Q-TOF-MS as described above. The control assay was carried out under the same conditions with heat-inactivated enzyme.

Reactions to determine the *K*_m_ of XimA toward xiamenmycin B contained 50 mM Tris-HCl buffer (pH 7.5), 5 mM MgSO_4_, 5 mM ATP, 10 mM L-threonine, 40 µM XimA and various concentrations of xiamenmycin B ranging from 0.2 µM to 345 µM. The reaction products were detected by Ultra Performance Liquid Chromatography and a Triple Quadrupole Mass Spectrometer (Waters ACQUITY UPLC, AB SCIEX SelexION Triple Quad 5500 System).

## Supporting Information

Figure S1 **^1^H NMR spectrum of xiamenmycin B.**
(TIF)

Figure S2 **^1^H-^1^H COSY spectrum of xiamenmycin B.**
(TIF)

Figure S3 **^13^C NMR spectrum of xiamenmycin B.**
(TIF)

Figure S4 **DEP-135 spectrum of xiamenmycin B.**
(TIF)

Figure S5 **HSGC spectrum of xiamenmycin B.**
(TIF)

Figure S6 **HMBC spectrum of xiamenmycin B.**
(TIF)

Figure S7 **NOE spectrum of xiamenmycin B.**
(TIF)

Figure S8 **Protein expression and purification.**
(TIF)

Figure S9 **Chemical structures of thirteen 4HB analogues.**
(TIF)

Figure S10 **Michaelis-Menten kinetics for activation of xiamenmycin B by XimA.**
(TIF)

Figure S11 **UPLC-extracted ion chromatography MS (EIC-MS) of XimD and XimE** ***in*** ***vitro*** **assays.**
(TIF)

Figure S12 **Gene replacement of** ***ximB.***
(TIF)

Figure S13 **Gene replacement of** ***ximC.***
(TIF)

Figure S14 **Gene replacement of** ***ximD.***
(TIF)

Figure S15 **Gene replacement of** ***ximE.***
(TIF)

Table S1 **Strains and plasmids used and generated in this study.**
(DOCX)

Table S2 **Primers used for construction and confirmation of mutants and for protein expression.**
(DOCX)

Table S3 **^1^H NMR data of compound 3.**
(DOCX)

Table S4 **^13^C NMR data of compound 3.**
(DOCX)
