# Supplementary figures and images for: Characterization of the Xiamenmycin Biosynthesis Gene Cluster in Streptomyces xiamenensis 318
Source: PLoS One. 2014 Jun 11;9(6):e99537. doi: 10.1371/journal.pone.0099537 (PMC4053376; doi:10.1371/journal.pone.0099537)

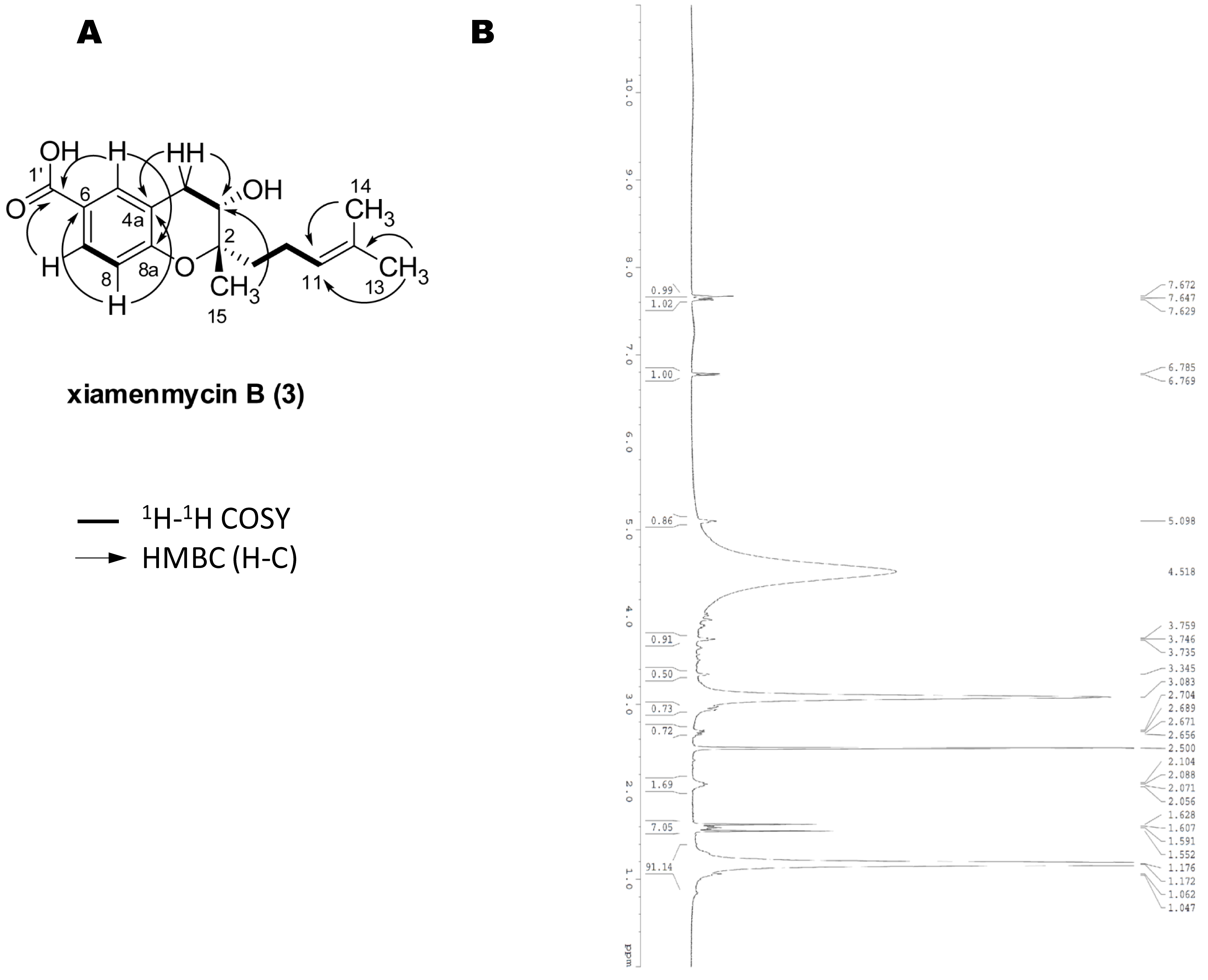

Supplement: Figure S1 — 1H NMR spectrum of xiamenmycin B. (TIF) [file pone.0099537.s001.tif]

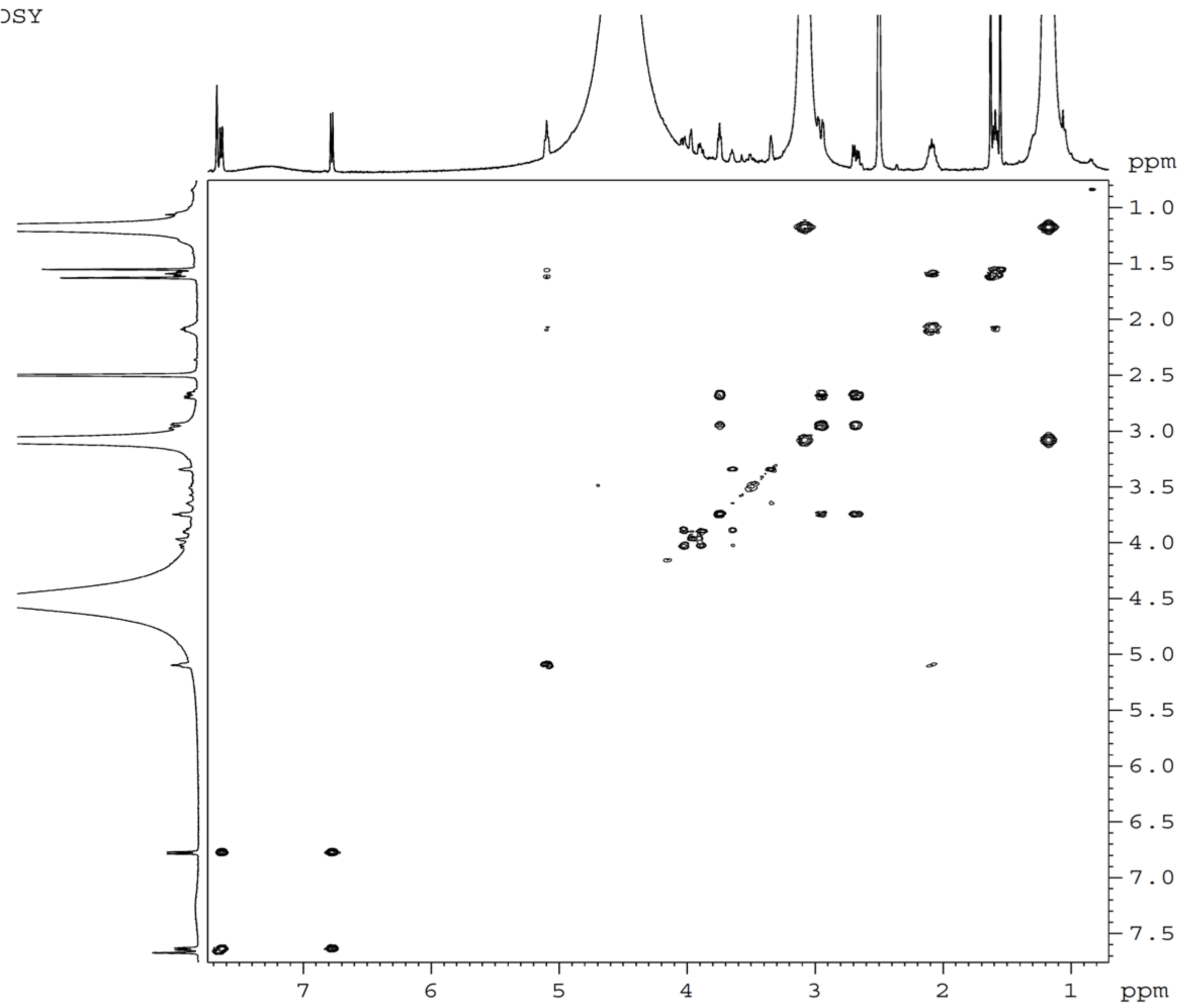

Supplement: Figure S2 — 1H-1H COSY spectrum of xiamenmycin B. (TIF) [file pone.0099537.s002.tif]

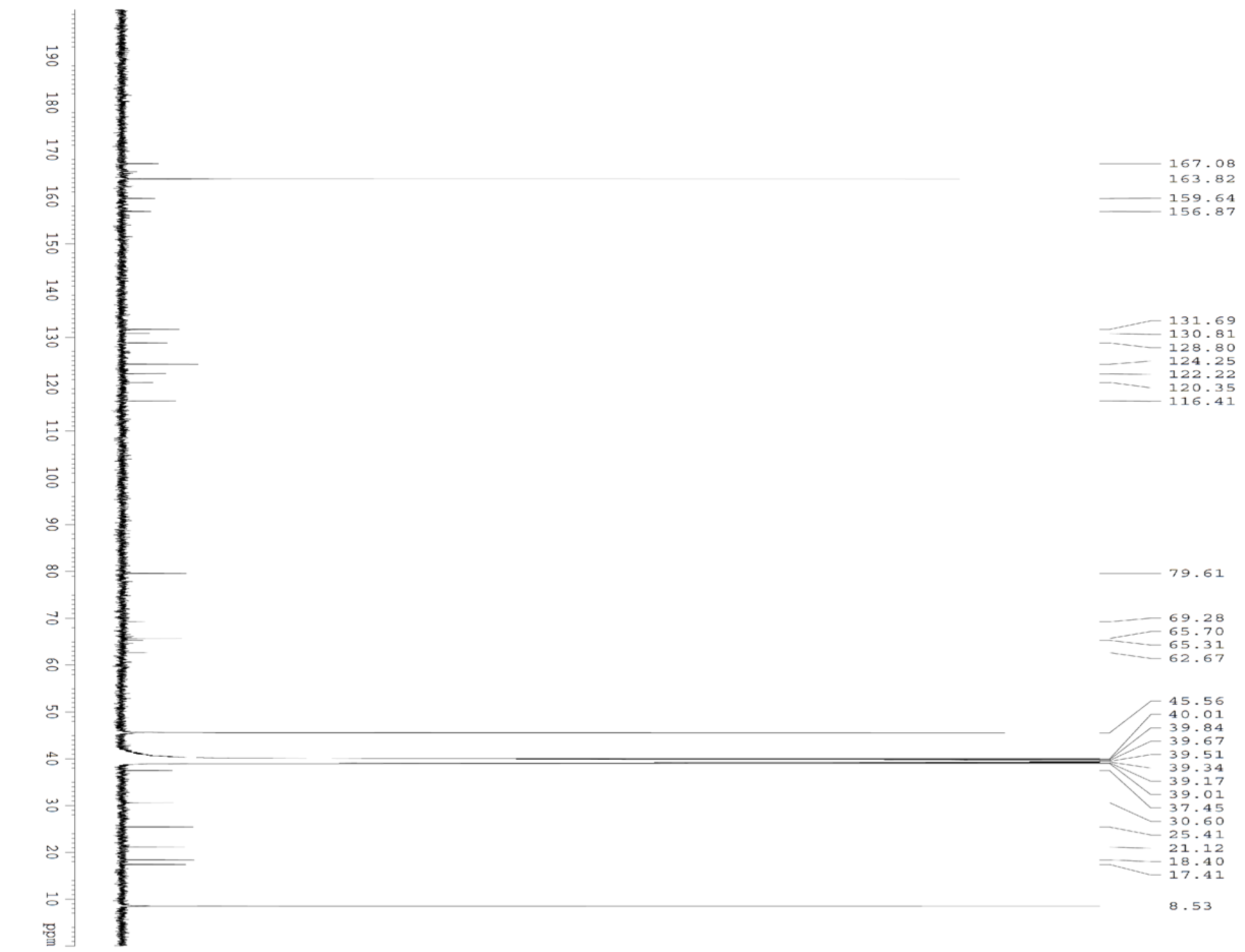

Supplement: Figure S3 — 13C NMR spectrum of xiamenmycin B. (TIF) [file pone.0099537.s003.tif]

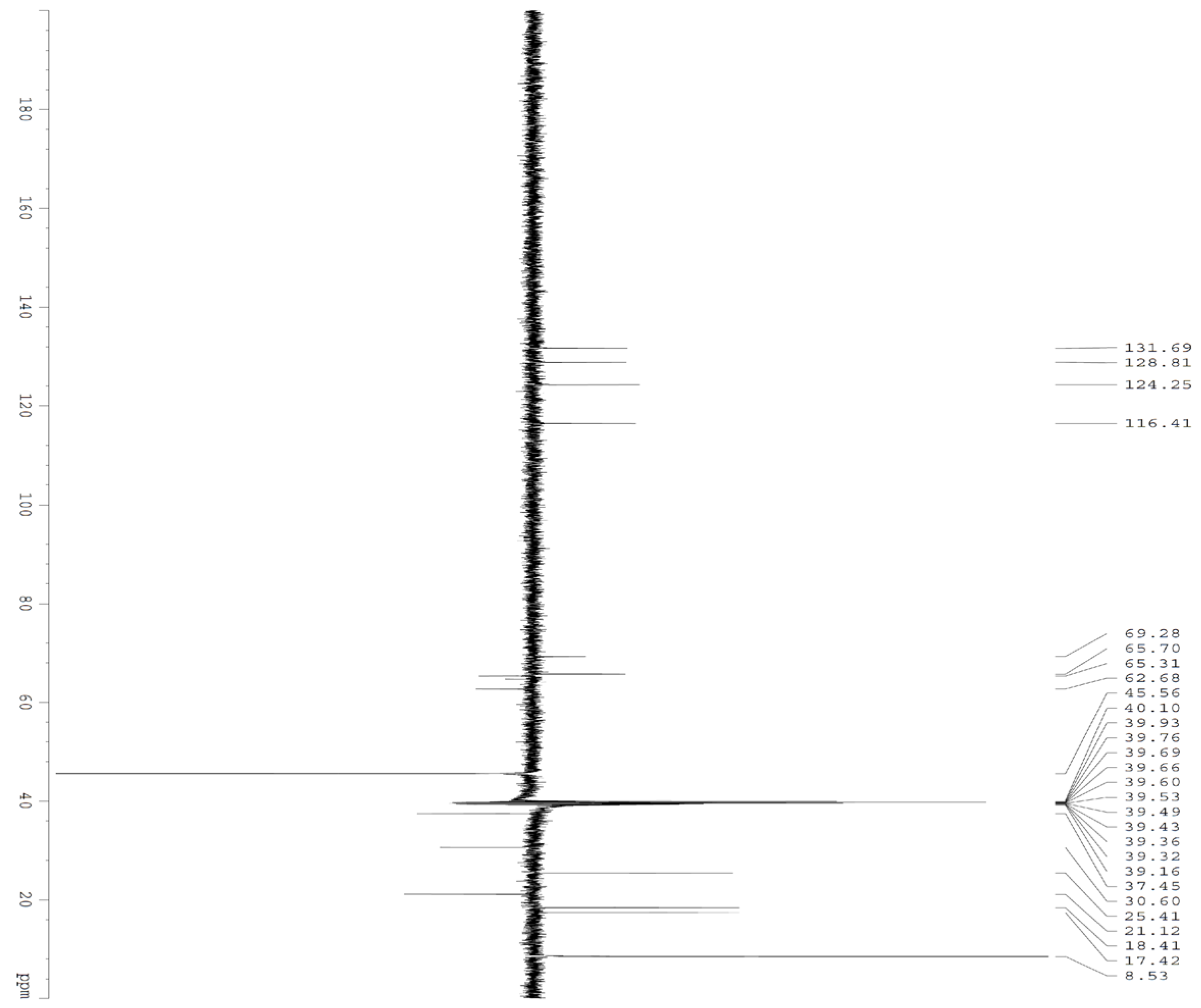

Supplement: Figure S4 — DEP-135 spectrum of xiamenmycin B. (TIF) [file pone.0099537.s004.tif]

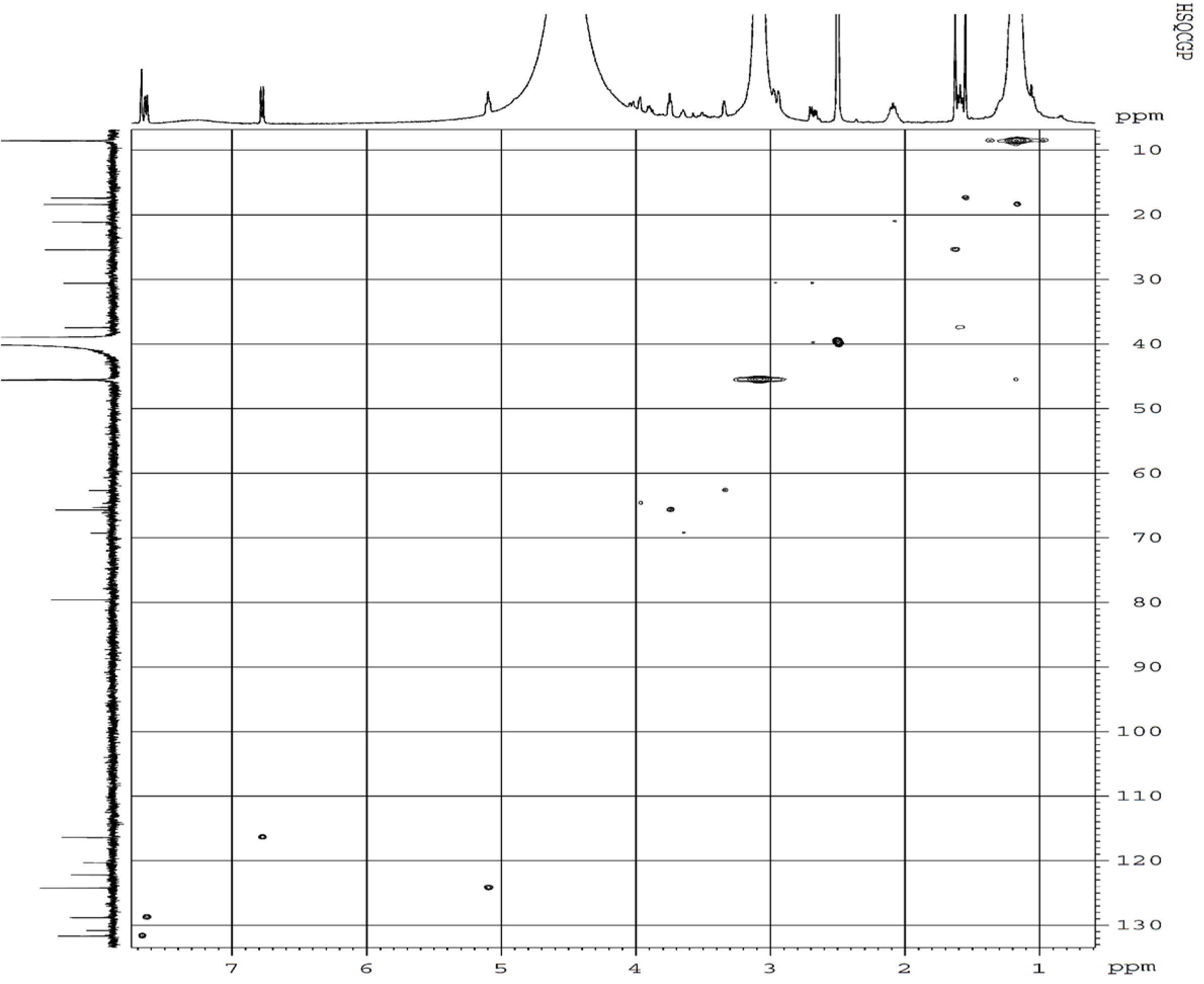

Supplement: Figure S5 — HSGC spectrum of xiamenmycin B. (TIF) [file pone.0099537.s005.tif]

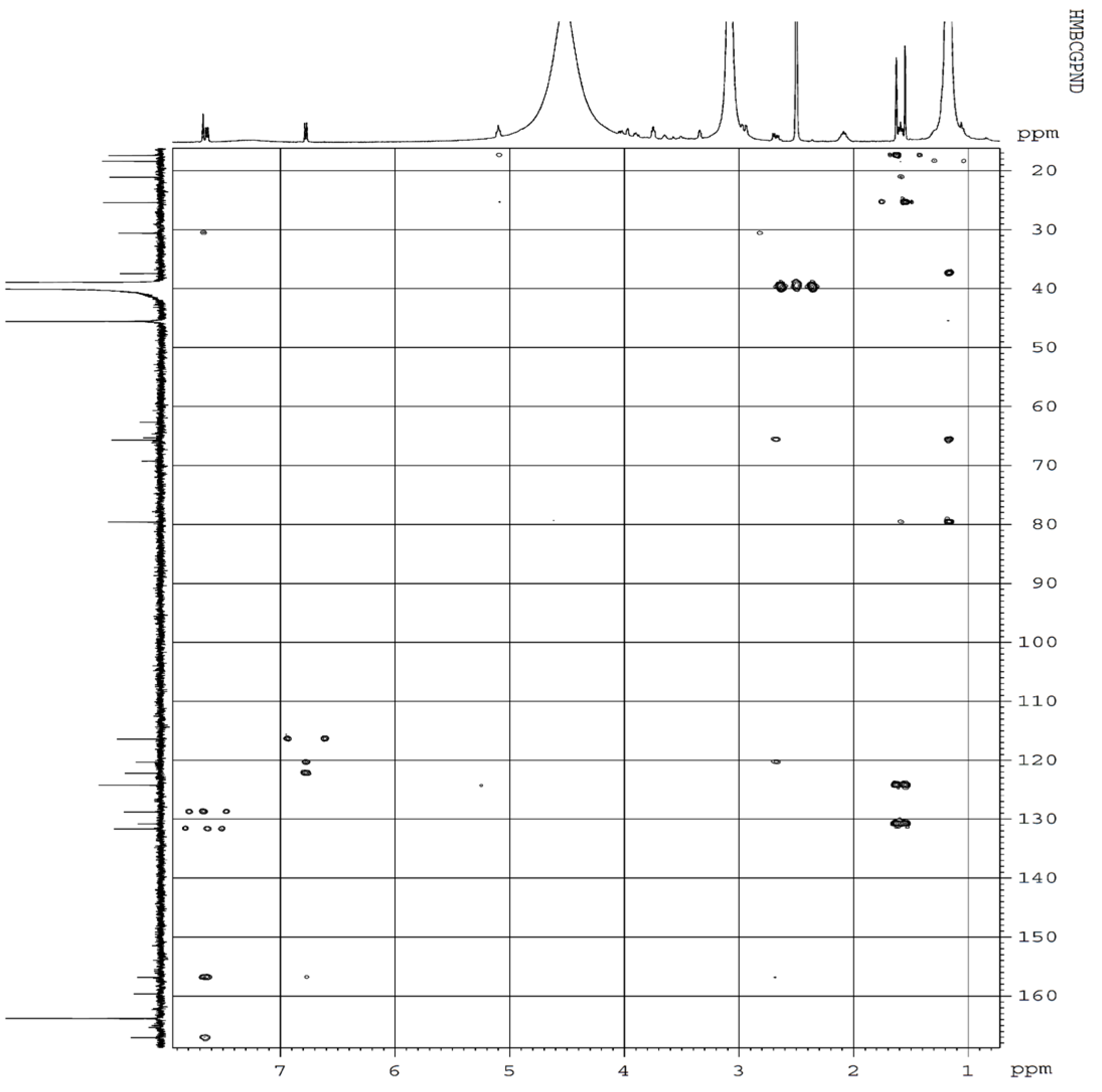

Supplement: Figure S6 — HMBC spectrum of xiamenmycin B. (TIF) [file pone.0099537.s006.tif]

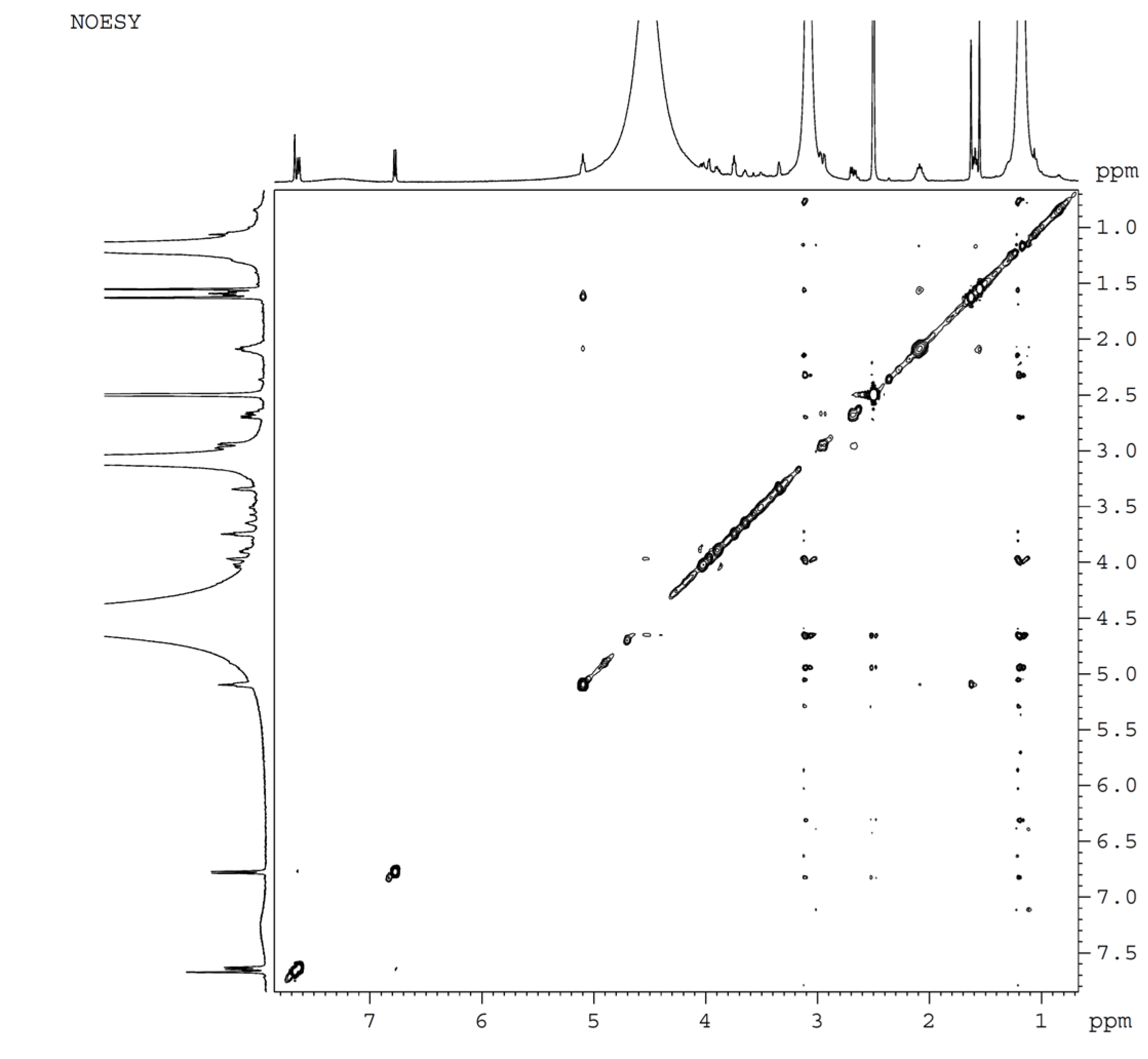

Supplement: Figure S7 — NOE spectrum of xiamenmycin B. (TIF) [file pone.0099537.s007.tif]

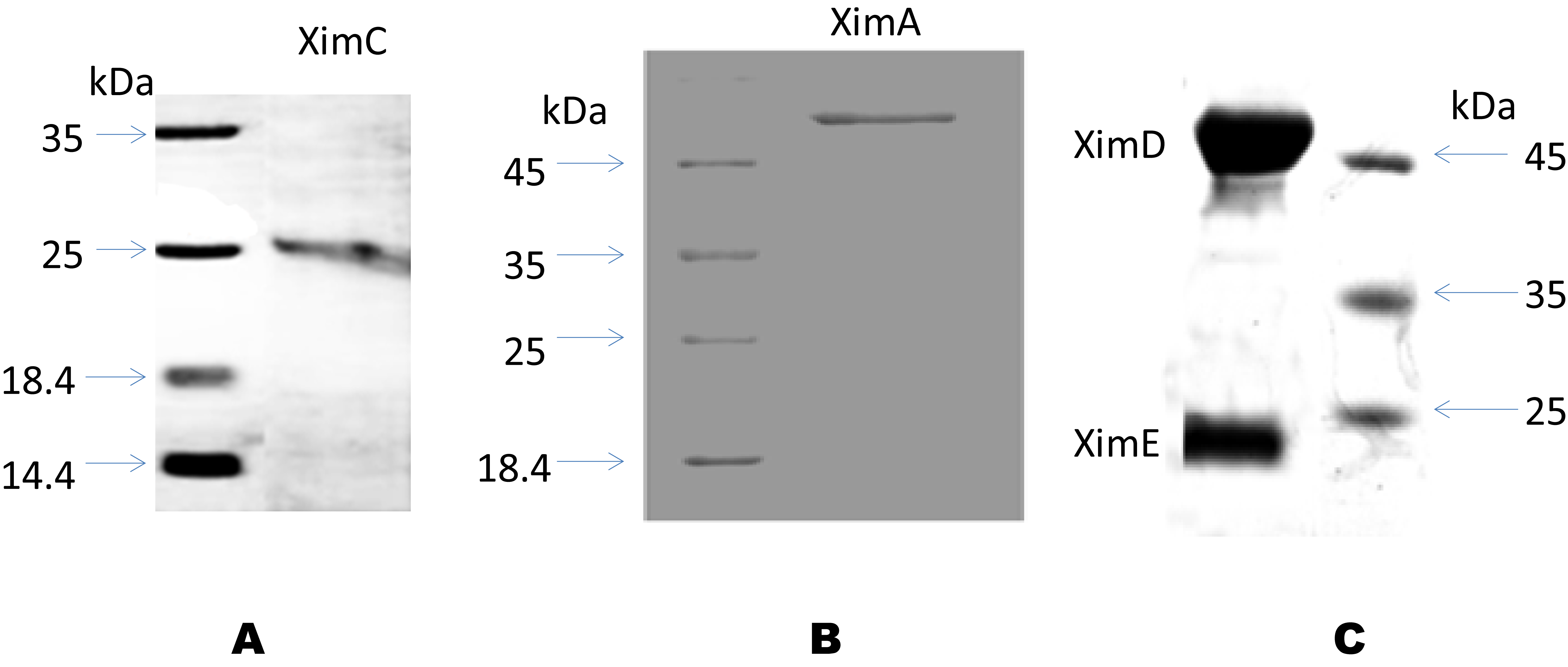

Supplement: Figure S8 — Protein expression and purification. (TIF) [file pone.0099537.s008.tif]

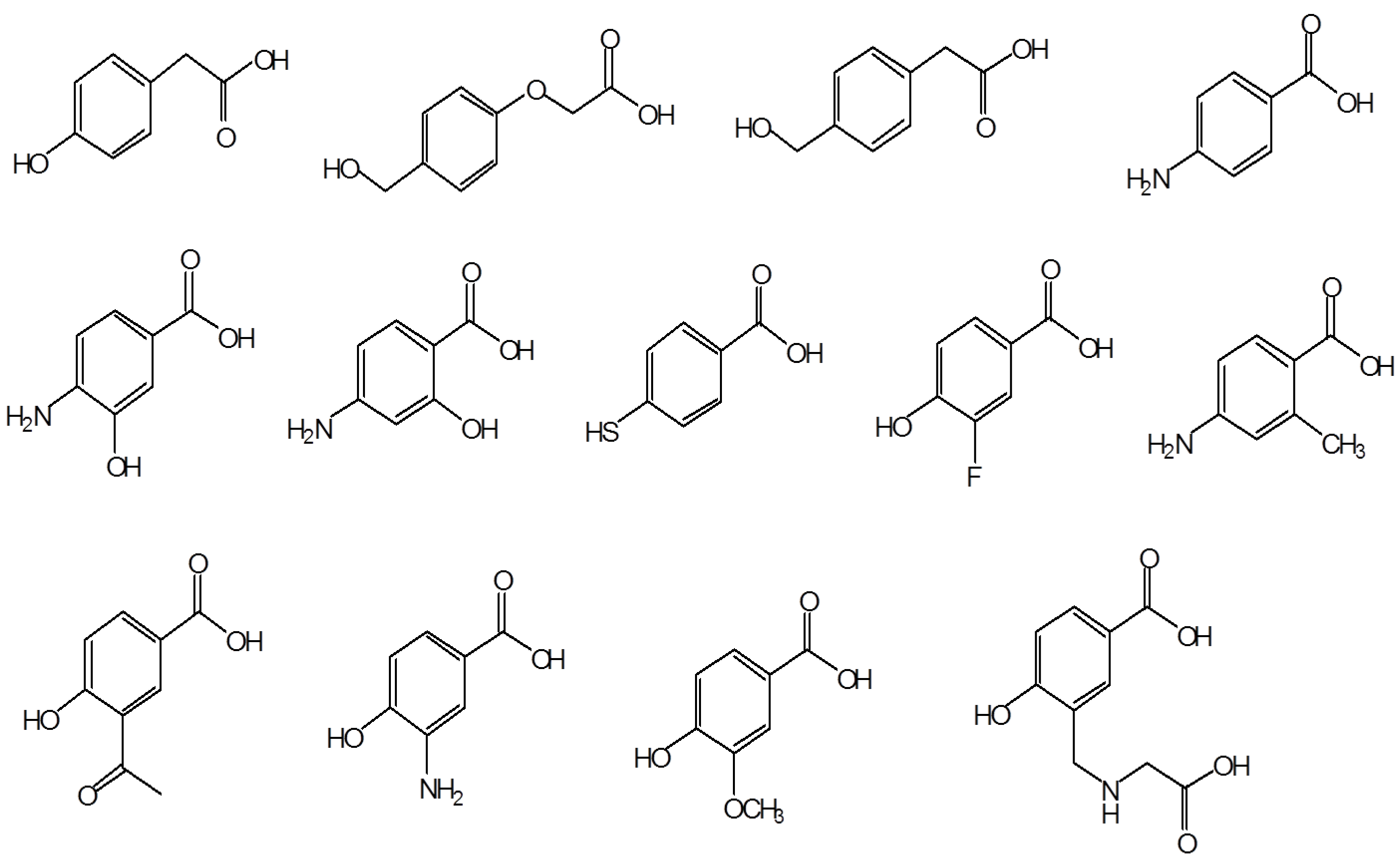

Supplement: Figure S9 — Chemical structures of thirteen 4HB analogues. (TIF) [file pone.0099537.s009.tif]

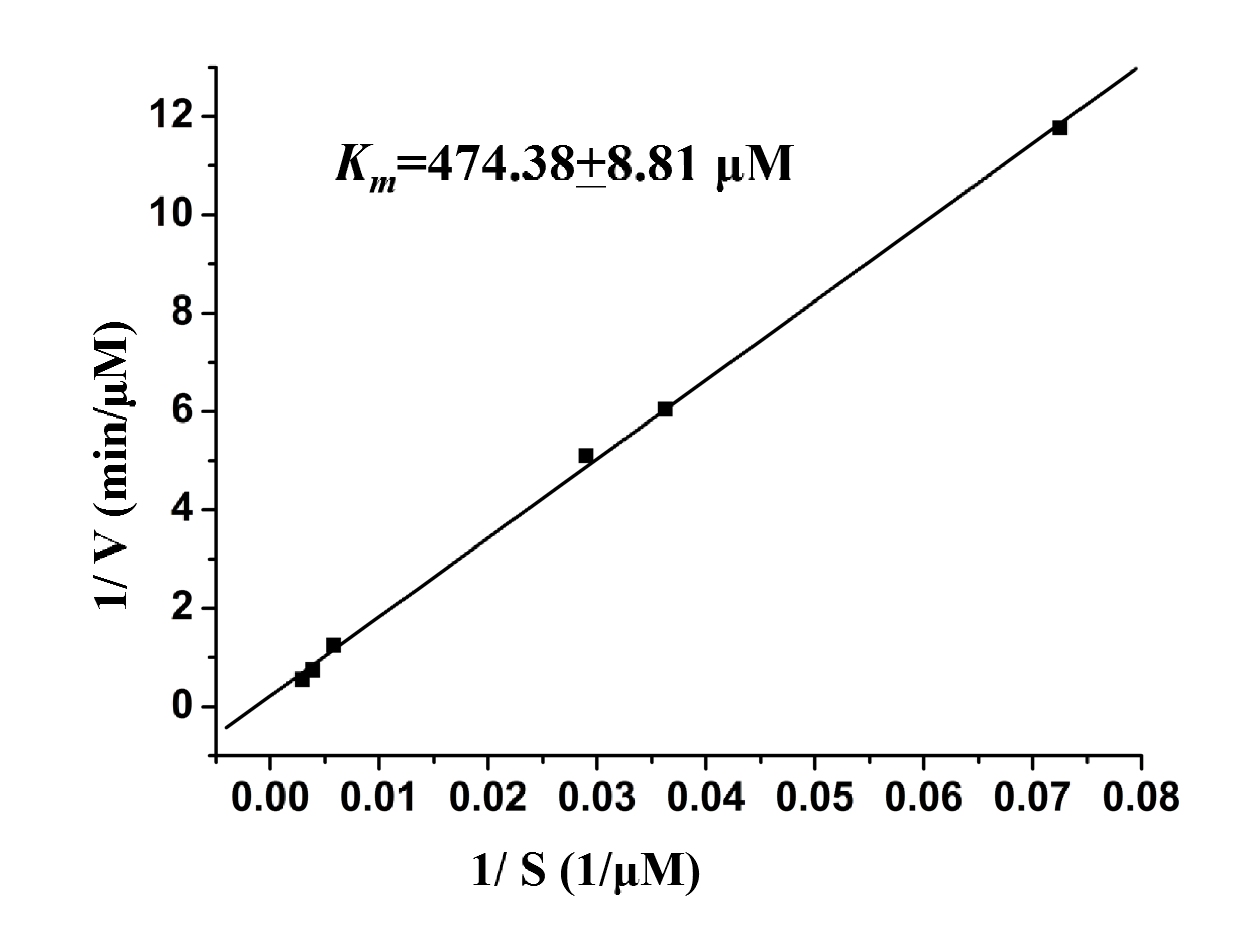

Supplement: Figure S10 — Michaelis-Menten kinetics for activation of xiamenmycin B by XimA. (TIF) [file pone.0099537.s010.tif]

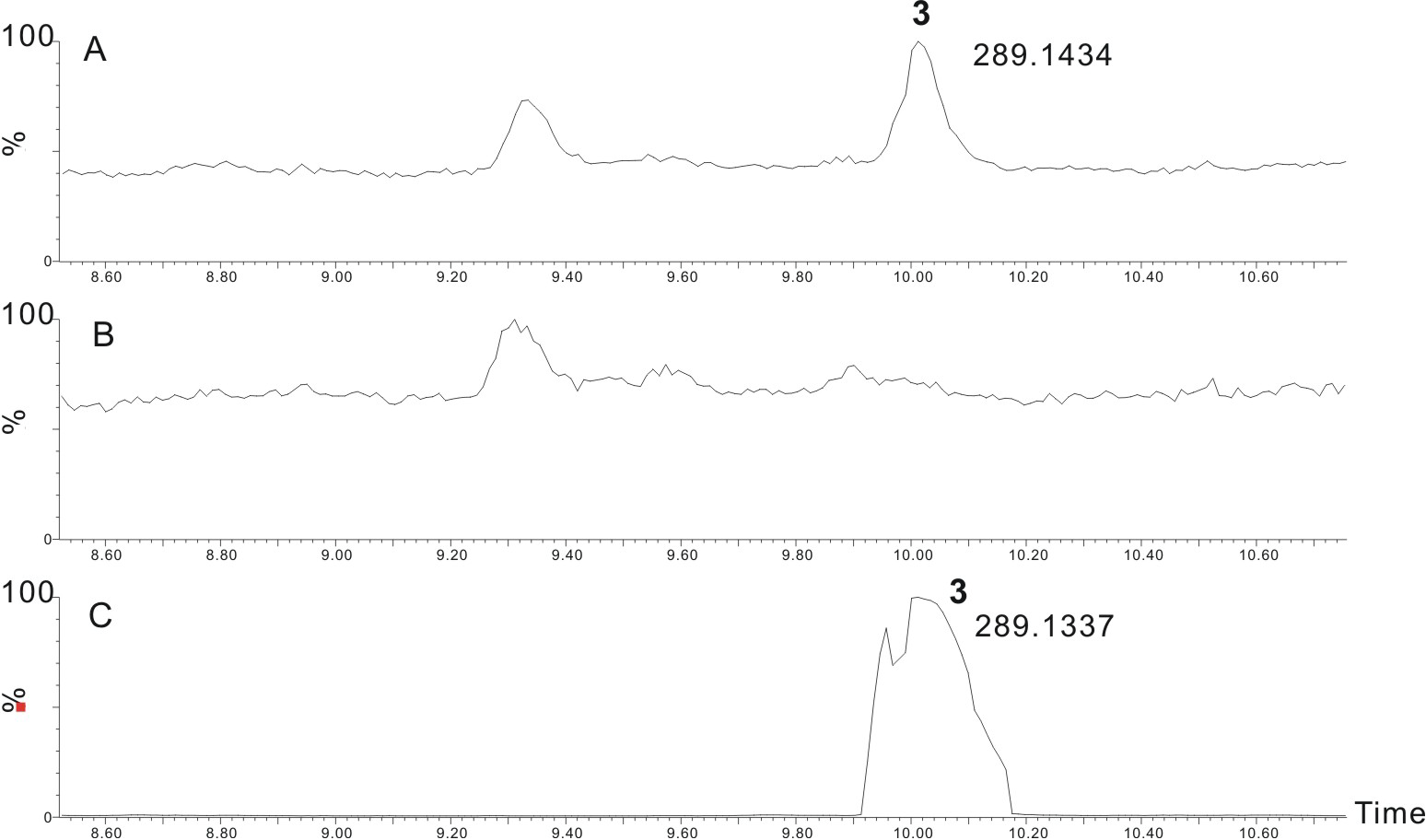

Supplement: Figure S11 — UPLC-extracted ion chromatography MS (EIC-MS) of XimD and XimE in vitro assays. (TIF) [file pone.0099537.s011.tif]

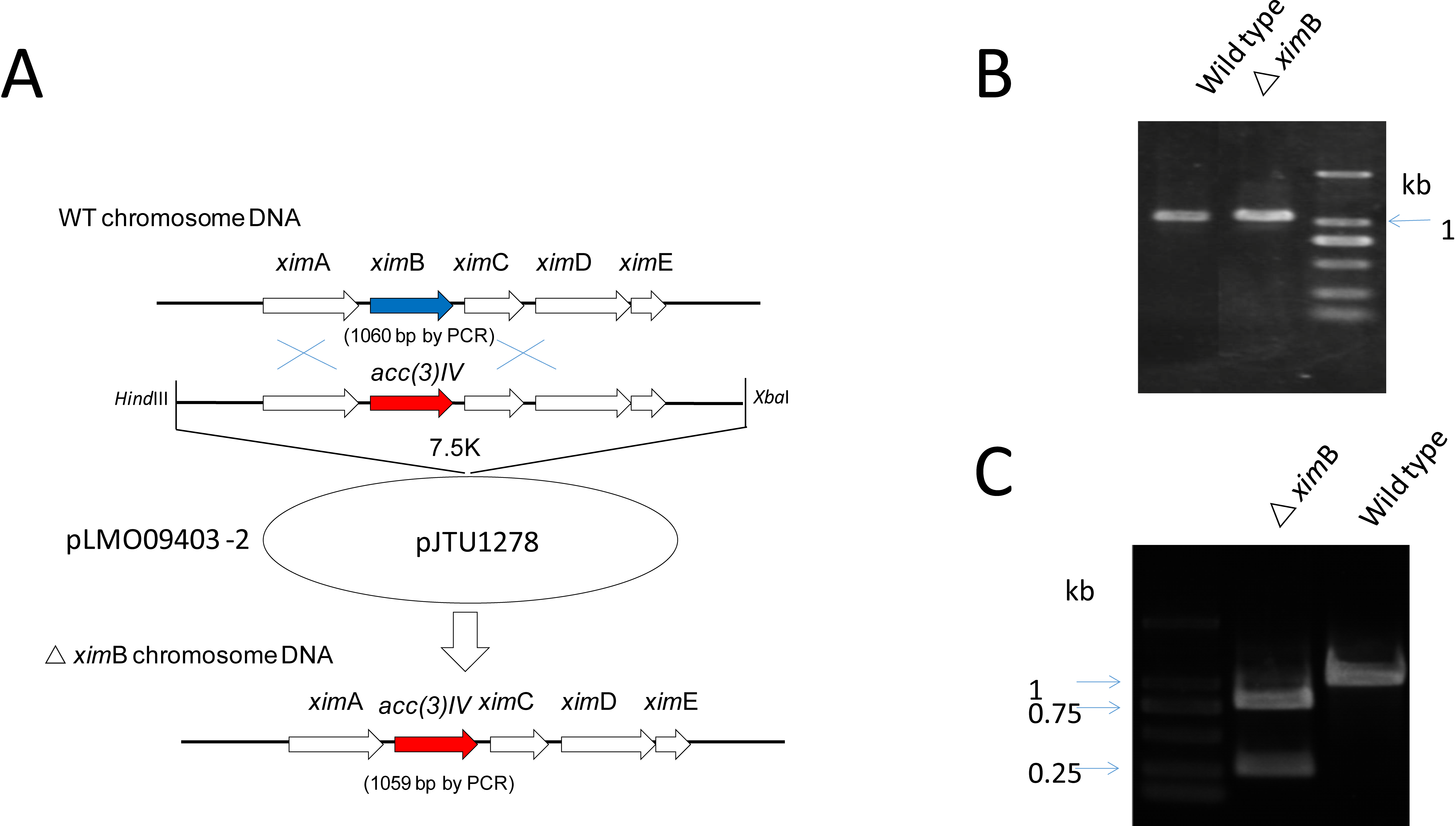

Supplement: Figure S12 — Gene replacement of ximB. (TIF) [file pone.0099537.s012.tif]

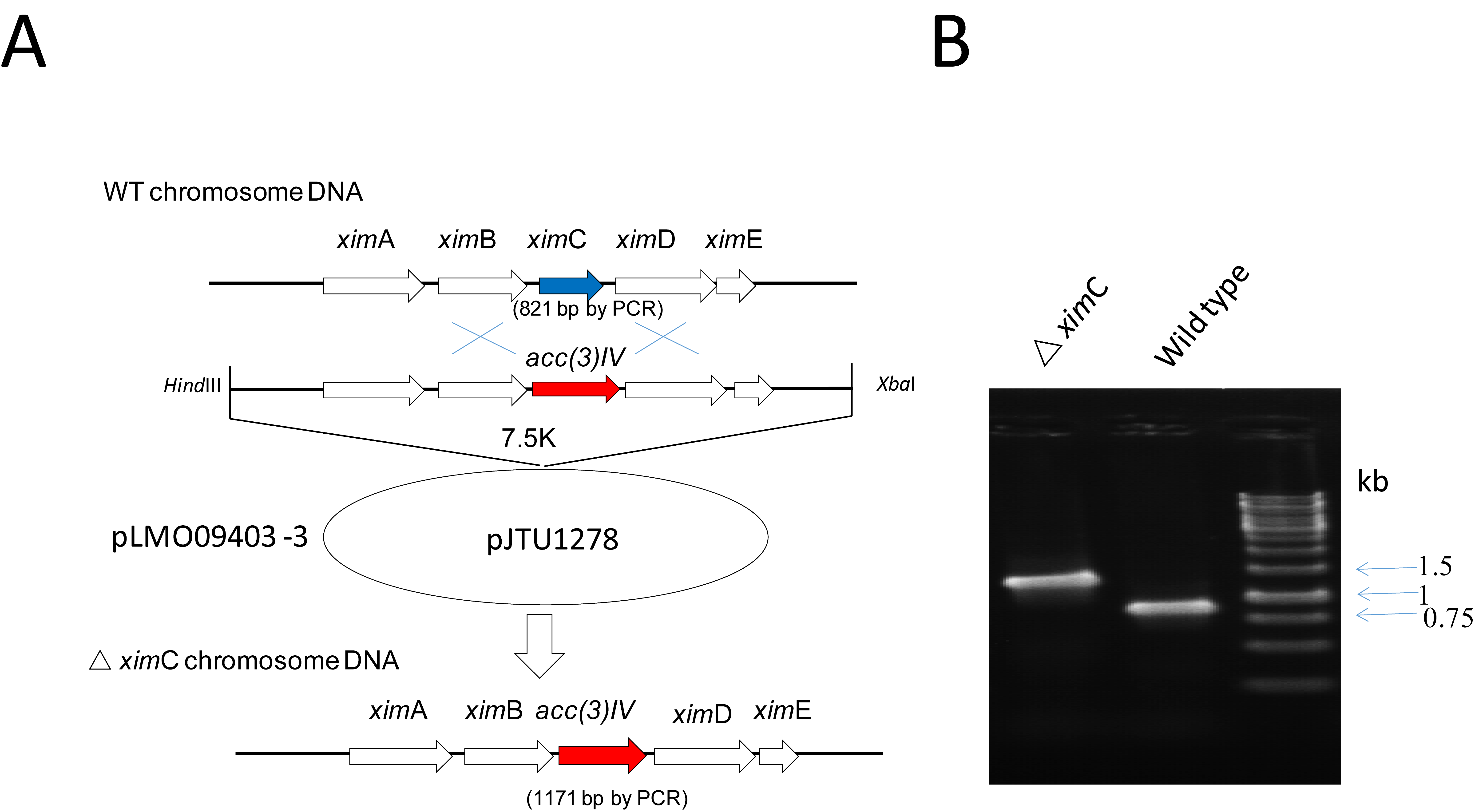

Supplement: Figure S13 — Gene replacement of ximC. (TIF) [file pone.0099537.s013.tif]

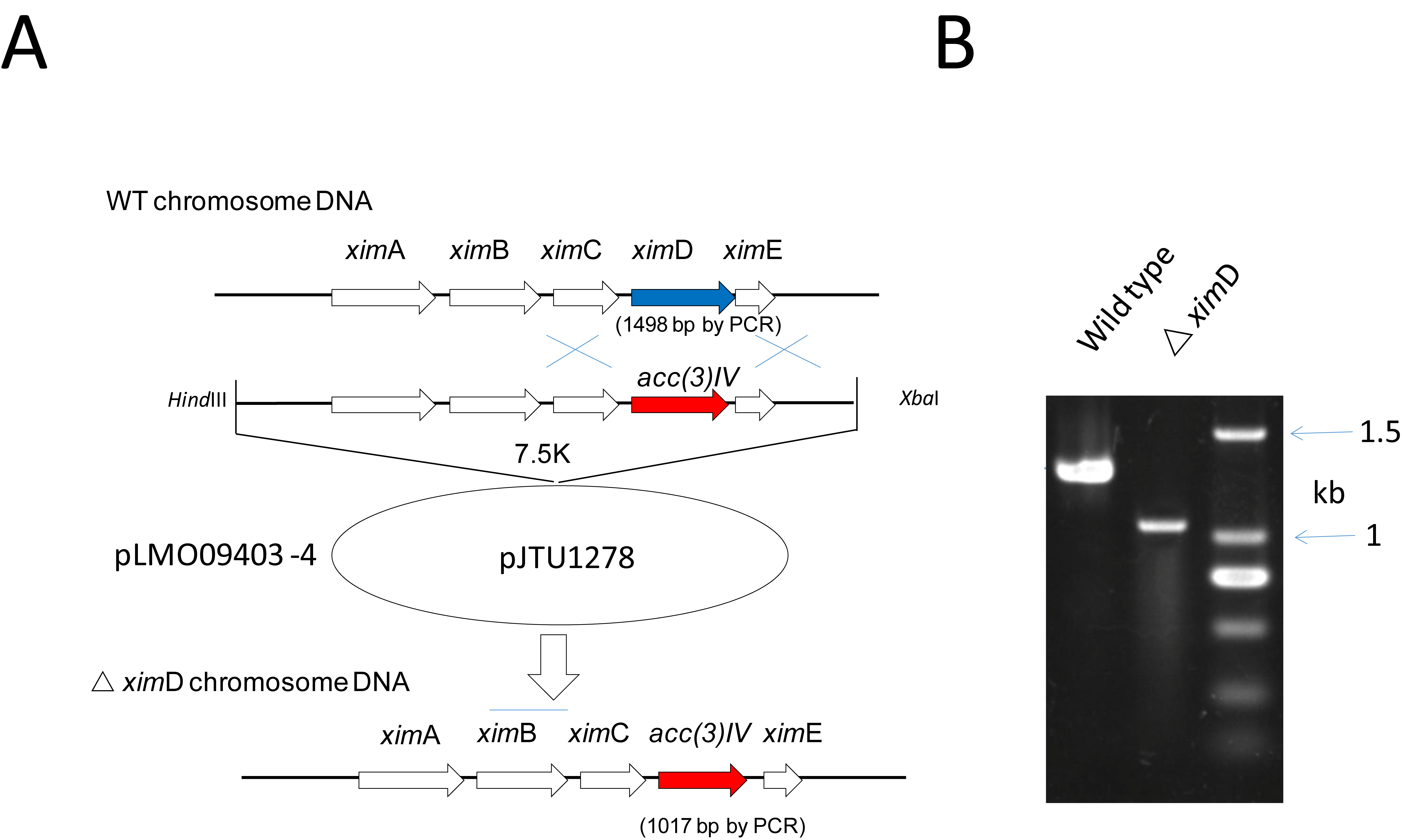

Supplement: Figure S14 — Gene replacement of ximD. (TIF) [file pone.0099537.s014.tif]

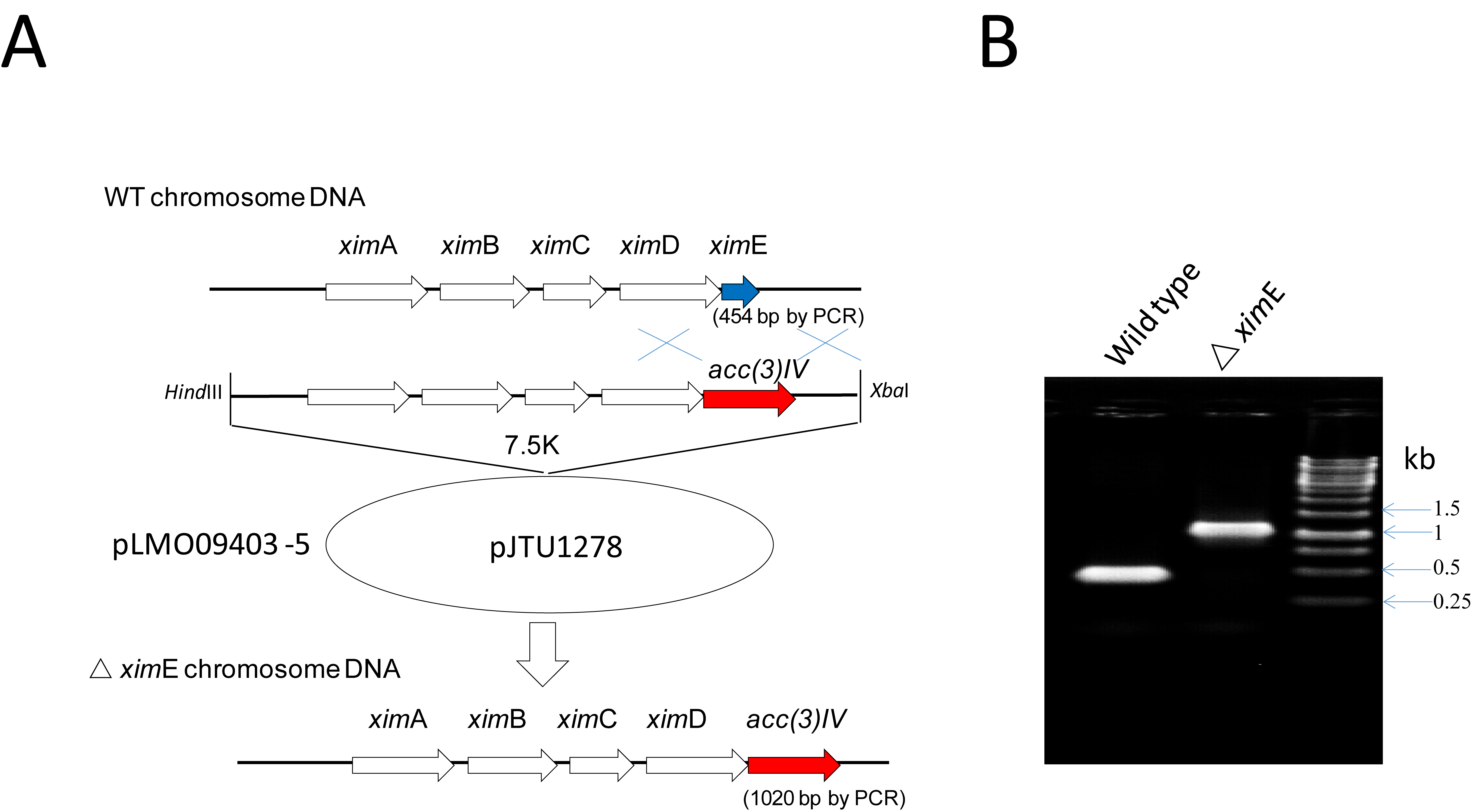

Supplement: Figure S15 — Gene replacement of ximE. (TIF) [file pone.0099537.s015.tif]
