# Supplementary material for: Characterization of the Xiamenmycin Biosynthesis Gene Cluster in Streptomyces xiamenensis 318
Source: PLoS One. 2014 Jun 11;9(6):e99537. doi: 10.1371/journal.pone.0099537 (PMC4053376; doi:10.1371/journal.pone.0099537)
Supplement: Table S1 — Strains and plasmids used and generated in this study. (DOCX) [file pone.0099537.s016.docx]

Table S1. Strains and plasmids used and generated in this study

| Strain/Plasmid | Purpose | Source |
| --- | --- | --- |
| Strains |  |  |
| *E. coli* |  |  |
| DH5α | Host strain for cloning | Invitrogen |
| BW25113 | Host strain for PCR targeting | Smanski et al. (2011) |
| ET12567 | Donor strain for conjugation | Li et al. (2008) |
| EPI300^TM^ | Host strain for genomic library | Epicentre |
| BL21(DE3) | Host strain for protein expression | Invitrogen |
| *S. lividans* |  |  |
| Strain 1326 | Host strain for heterologous expression |  |
| *S. xiamenensis* |  |  |
| Strain 318 | Xiamenmycin producing strain | Xu et al. (2009) |
| *xim*A | *xim*A inactivation mutant of strain 318 | This study |
| *xim*B | *xim*B inactivation mutant of strain 318 | This study |
| *xim*C | *xim*C inactivation mutant of strain 318 | This study |
| *xim*D | *xim*D inactivation mutant of strain 318 | This study |
| *xim*E | *xim*E inactivation mutant of strain 318 | This study |
| Plasmids |  |  |
| pMD18-T | Amp^r^. vector for cloning | TAKARA |
| pET28a+ | Kan^r^. vector for protein expression | Novagen |
| pET Duet-1 | Amp^r^. vector for protein coexpression | Novagen |
| pCC2FOS^TM^ | Fosmid vector | Epicentre |
| pJTU1278 | Amp^r^. Tsr^r^. vector for PCR Targeting and conjugation | He et al. (2010) |
| pSET152 | Apr^r^. vector for heterologous expression | Flett et al. (1997) |
| p9A11 | Chl^r^. vector for amplifying genes | This study |
| pLMO09403 | Amp^r^. Tsr^r^. 7.5 kb *Hind*III-*Xba*I fragment cloned in pJTU1278, containing *xim* gene cluster | This study |
| pLMO09403-1 | Amp^r^. Tsr^r^. Apr^r^.subclone for *xim*A inactivation | This study |
| pLMO09403-2 | Amp^r^. Tsr^r^. Apr^r^.subclone for *xim*B inactivation | This study |
| pLMO09403-3 | Amp^r^. Tsr^r^. Apr^r^. subclone for *xim*C inactivation | This study |
| pLMO09403-4 | Amp^r^. Tsr^r^. Apr^r^.subclone for *xim*D inactivation | This study |
| pLMO09403-5 | Amp^r^. Tsr^r^. Apr^r^.subclone for *xim*E inactivation | This study |
| pLMO09404 | Apr^r^. 7.8 kb *Eco*RI-*Xba*I fragment cloned in pSET152, containing *xim* gene cluster for heterologous expression | This study |
| pLMO09405 | Kan^r^. protein expression clone for *xim*A expression | This study |
| pLMO09406 | Kan^r^. protein expression clone for *xim*B expression | This study |
| pLMO09407 | Kan^r^. protein expression clone for *xim*C expression | This study |
| pLMO09408 | Amp^r^. protein expression clone for *xim*D and *xim*E coexpression | This study |
| pLMO09409 | Amp^r^. protein expression clone for *xim*D expression | This study |
| pLMO09410 | Kan^r^. protein expression clone for *xim*E expression | This study |

Amp^r^, ampicillin resistance; Tsr^r^, thiostrepton resistance; Apr^r^, apramycin resistance.

Reference：

Li, L., Xu, Z., Xu, X., Wu, J., Zhang, Y., He, X., Zabriskie, T. M., Deng, Z. (2008). The mildiomycin biosynthesis: initial steps for sequential generation of 5-hydroxymethylcytidine 5'-monophosphate and 5-hydroxymethylcytosine in *Streptoverticillium rimofaciens* ZJU5119. Chembiochem 9, 1286.

Smanski, M. J., Yu, Z., Casper, J., Lin, S., Peterson, R. M., Chen, Y., Wendt-Pienkowski, E., Rajski, S. R., Shen, B. (2011). Dedicated ent-kaurene and ent-atiserene synthases for platensimycin and platencin biosynthesis. *Proceedings of the National Academy of Sciences* *108*, 13498.

He, Y., Wang, Z., Bai, L., Liang, J., Zhou, X., and Deng, Z. (2010). Two pHZ1358-derivative vectors for efficient gene knockout in *streptomyces*. Journal of microbiology and biotechnology 20, 678-682.

Flett, F., Mersinias, V., Smith, C. P. (1997). High efficiency intergeneric conjugal transfer of plasmid DNA from *Escherichia coli* to methyl DNA-restricting *streptomycetes*. FEMS Microbiol Lett 155, 223-229.

Xu, J., Wang, Y., Xie, S.J., Xiao, J., and Ruan, J.S. (2009). *Streptomyces xiamenensis* sp. nov., isolated from mangrove sediment. Int J Syst Evol Microbiol 59, 472-476.
