# Supplementary material for: Characterization of the Xiamenmycin Biosynthesis Gene Cluster in Streptomyces xiamenensis 318
Source: PLoS One. 2014 Jun 11;9(6):e99537. doi: 10.1371/journal.pone.0099537 (PMC4053376; doi:10.1371/journal.pone.0099537)
Supplement: Table S2 — Primers used for construction and confirmation of mutants and for protein expression. (DOCX) [file pone.0099537.s017.docx]

Table S2. Primers used for constructing and confirming the mutants and for protein expression

| Primer | Gene | Sequence(5‘-3‘) |
| --- | --- | --- |
| **For screening library** |  |  |
| ORF5317-For  ORF5317-Rev | ORF5317 | GCGTGCGGACATGCGTGTTGTGGTG  GGGTCCAACCTACCCAAATGTGATGGAGCAA |
| ORF5310-For  ORF5310-Rev | ORF5310 | GCGGCTGGAGTGTAGCGAGTCTGGAATG  GGGTATGTCCACCCGATGCTCCTGTCTCAT |
| **For PCR Targeting** |  |  |
| TORF5311-For  TORF5311-Rev | *ximA* | ATGAGACAGGAGCATCGGGTGGACATACCCGAGAACTTGTGGTTCATGTGCAGCTCCATC  TCACGTTCGAGGCGCATTCGACGCCGGATAGTGACGATG TGAGCTCAGCCAATCGACTG |
| TORF5313-For  TORF5313-Rev | *ximB* | GTGATCGATATTTCCGCTCAACCCTCGCAGCAGAGCACGTGGTTCATGTGCAGCTCCATC  TCAAAAGACTCTCCCCGCAACGATGGCGACGAGCACGAGTGAGCTCAGCCAATCGACTG |
| TORF5314-For  TORF5314-Rev | *ximC* | GTGCGCACGGAGTCGCGCAGCCTGGCCCAGTTCGTGGCGTGGTTCATGTGCAGCTCCATC  TCATGCGTCGTGGACGGCGTCTCGATCGAGGAGACACGGTGAGCTCAGCCAATCGACTG |
| TORF5315-For  TORF5315-Rev | *ximD* | ATGCCGAACTCTCCCGCCGCGGTCTTCGAGCGGCTCACCTGGTTCATGTGCAGCTCCATC  TCACGTCGTCTCCATCATCGTGTACTCCTGCCGGATCCGTGAGCTCAGCCAATCGACTG |
| TORF5316-For  TORF5316-Rev | *ximE* | ATGGGCCAGACGACGCACACAGCACTCGACCGCTACATGTGGTTCATGTGCAGCTCCATC  TCAGCCCGGCGTACGGGTGTACCGGTTGCGCAGGTTCGTTGAGCTCAGCCAATCGACTG |
| **For gene verification** |  |  |
| VORF5311-For  VORF5311-Rev | *ximA* | GCCCGAACCCCTGAGACTCTGGAGATC  GCCGCCTTGTCCTAACGGTGTTTGCC |
| VORF5313-For  VORF5313-Rev | *ximB* | CCGAGGTGGGCTTTCTGCAAGAGTCA  GCACGGACCAGGGGCCTGCAT |
| VORF5314-For  VORF5314-Rev | *ximC* | CTGCCGGGAAAAGTTCATGGCAAACCG  TGCCGAGACGAACCTCGATGCGGAC |
| VORF5315-For  VORF5315-Rev | *ximD* | GCCGTCCACGACGCATGAGGGATC  GGTCGGCGAGCTCCATGTAGCGGTC |
| VORF5316-For  VORF5316-Rev | *ximE* | CGGCAGGAGTACACGATGATGGAGACG  TCTCGGCAGCTACCGGCGCATC |
| **For heterologous expression** |  |  |
| HORF5311-6-For  HORF5311-6-Rev | *ximA~ximE* | GCTCTAGACGGCTGGAGTGTAGCGAGTCTGGAATG  GGAATTCCCCGGACGTGGGAGCGATAGGG |
| **For protein expression** |  |  |
| *xim*A-F-NdeⅠ  *xim*A-R-XhoⅠ | *ximA* | TCAGCcatATGAGACAGGAGCATCGGGTGG  TCAGctcgagTCACGTTCGAGGCGCATTC |
| *xim*B-F-NdeⅠ  *xim*B-R-XhoⅠ | *ximB* | ctagcataTGGTGATCGATATTTCCGCTC  atcgctcgagTCAAAAGACTCTCCCCGCAAC |
| *xim*C-F-NdeⅠ  *xim*C-R-HindⅢ | *ximC* | ataccataTGCGCACGGAGTCG  atacaagcttTCATGCGTCGTGGACG |
| *xim*D-F-EcoRⅠ  *xim*D-R-HindⅢ | *ximD* | TCAGgaattcATGCCGAACTCTCCCGC  TCAGaagcttTCACGTCGTCTCCATCATCG |
| *xim*E-F-NdeⅠ  *xim*E-R- HindⅢ | *ximE* | TCGAcatATGGGCCAGACGACGCACAC  TCAGaagcttTCAGCCCGGCGTACGGGT |
| **For protein coexpression** |  |  |
| *xim*D-F-EcoRⅠ  *xim*D-R-HindⅢ | *ximD* | TCAGgaattcATGCCGAACTCTCCCGC  TCAGaagcttTCACGTCGTCTCCATCATCG |
| Co*xim*E-F-NdeⅠ  Co*xim*E-R-KpnⅠ | *ximE* | TCGAcatATGGGCCAGACGACGCACAC  TAGCggtaccTCAGCCCGGCGTACGGGT |
| **For RT-PCR** |  |  |
| ORF1467 | ORF1467 | GCCAACGCCCCCTCACCTC  GCCGACCACCATCATGCTC |
| ORF2290 | ORF2290 | GCGTCGGCGAAGTCGTTGA  GCGTCCGTCTGCCTCTACC |
| ORF2781 | ORF2781 | ACCTCGGCACCCGCACCAC  CGGCCACCAGTCCCACTCC |
| ORF4925 | ORF4925 | ACCGTCACCCCGCACAAGT  GCCATCCAGCAGAAGAACA |
| ORF5065 | ORF5065 | ACGTGGTGATGTTCGAGCA  GTCAGGGAGGCGGTGTAGG |
| ORF5313 | ORF5313 | TGGCTGGGGATGGTCTTCG  CCTTGTCCTGGTGGGCGTA |
