# Supplementary material for: Characterization of the Xiamenmycin Biosynthesis Gene Cluster in Streptomyces xiamenensis 318
Source: PLoS One. 2014 Jun 11;9(6):e99537. doi: 10.1371/journal.pone.0099537 (PMC4053376; doi:10.1371/journal.pone.0099537)
Supplement: Table S3 — 1H NMR data of compound 3. (DOCX) [file pone.0099537.s018.docx]

Table S3. ^1^H NMR data of compound **3** (in DMSO-d_6_, 500 MHz Bruker Avance III)

| Site | **1*** | **3** |
| --- | --- | --- |
| 1 | - | - |
| 2 | - | - |
| 3 | 3.77,t | 3.75, t |
| 4 | 2.71, dd (17.3, 7.4)  2.98, dd (17.3, 5.2) | 2.68, dd (17.0, 7.5)  2.94, dd (17.0, 5.5) |
| 4a | - | - |
| 5 | 7.67, s | 7.67, s |
| 6 | - | - |
| 7 | 7.63, d (8.4) | 7.64, d (8.5) |
| 8 | 6.81, d (8.4) | 6.78, dd (8.5, 1.5) |
| 8a | - | - |
| 9 | 1.60, m | 1.60, m |
| 10 | 2.10, m | 2.08, m |
| 11 | 5.12, dd (7.1, 1.3) | 5.10, t (7.5) |
| 12 | - | - |
| 13 | 1.57, s | 1.55, s |
| 14 | 1.65, s | 1.63, s |
| 15 | 1.18, s | 1.18, s |
| 1’ | - | - |
| 2’ | 7.78, d (7.8) | - |
| 3’ | 4.38, brd | - |
| 4’ | 4.18, brs | - |
| 5’ | 1.12, d (6.0) | - |
| 6’ | - | - |

*****See Reference:

Xu, M.J., Liu, X.J., Zhao, Y.L., Liu, D., Xu, Z.H., Lang, X.M., Ao, P., Lin, W.H., Yang, S.L., Zhang, Z.G., et al. (2012). Identification and characterization of an anti-fibrotic benzopyran compound isolated from mangrove-derived *Streptomyces xiamenensis*. Mar Drugs 10, 639-654.
