# Supplementary material for: Characterization of the Xiamenmycin Biosynthesis Gene Cluster in Streptomyces xiamenensis 318
Source: PLoS One. 2014 Jun 11;9(6):e99537. doi: 10.1371/journal.pone.0099537 (PMC4053376; doi:10.1371/journal.pone.0099537)
Supplement: Table S4 — 13C NMR data of compound 3. (DOCX) [file pone.0099537.s019.docx]

Table S4. ^13^C NMR data of compound **3** (in DMSO-d_6_, 500 MHz Bruker Avance III)

| Position | **1*** | **3** |
| --- | --- | --- |
| 1 | - | - |
| 2 | 79.8 | 79.6 |
| 3 | 66.3 | 65.7 |
| 4 | 31.2 | 30.6 |
| 4a | 120.6 | 120.4 |
| 5 | 129.8 | 131.7 |
| 6 | 126.0 | 122.3 |
| 7 | 127.2 | 128.8 |
| 8 | 116.7 | 116.4 |
| 8a | 156.1 | 156.9 |
| 9 | 38.0 | 37.5 |
| 10 | 21.6 | 21.1 |
| 11 | 124.8 | 124.3 |
| 12 | 131.3 | 130.8 |
| 13 | 17.9 | 17.4 |
| 14 | 25.9 | 25.4 |
| 15 | 18.7 | 18.4 |
| 1’ | 166.6 | 167.1 |
| 2’ | - | - |
| 3’ | 58.9 | - |
| 4’ | 67.1 | - |
| 5’ | 20.9 | - |
| 6’ | 172.8 | - |

*****See Reference:

Xu, M.J., Liu, X.J., Zhao, Y.L., Liu, D., Xu, Z.H., Lang, X.M., Ao, P., Lin, W.H., Yang, S.L., Zhang, Z.G., et al. (2012). Identification and characterization of an anti-fibrotic benzopyran compound isolated from mangrove-derived *Streptomyces xiamenensis*. Marine Drugs 10, 639-654.
